# CircSCAF8 promotes growth and metastasis of prostate cancer through the circSCAF8-miR-140-3p/miR-335-LIF pathway

**DOI:** 10.1038/s41419-022-04913-7

**Published:** 2022-06-02

**Authors:** Tao He, Wen Tao, Lei-Lei Zhang, Bang-Yu Wang, Ke Li, Hui-Min Lu, Guo-Jun Tang, Ya-Di He, Liao-Yuan Li

**Affiliations:** 1 Department of Urology, The Third Affiliated Hospital, Sun Yat-sen University, Guangzhou, 510630 China; 2 Department of Breast Surgery, The Third Affiliated Hospital, Sun Yat-sen University, Guangzhou, 510630 China; 3 Health Management Center, The Third Affiliated Hospital, Sun Yat-sen University, Guangzhou, 510630 China

**Keywords:** Small RNAs, Prostate cancer, Diagnostic markers

## Abstract

Circular RNAs (circRNAs) have been increasingly linked to cancer progression. However, the detailed biological functions of circRNAs in prostate cancer (PCa) remain unclear. Using high-throughput circRNA sequencing, we previously identified 18 urine extracellular vesicle circRNAs that were increased in patients with PCa compared with those with benign prostatic hyperplasia. Spearman correlation analysis of the expression levels of the 18 circRNAs between the tumor tissue and matched urine extracellular vesicles in 30 PCa patients showed that circSCAF8 had the highest *R*^2^ (*R*^2^ = 0.635, *P* < 0.001). The Cox proportional hazards regression model was used to estimate the effect of circSCAF8 on progression-free survival. The in vitro and in vivo functional experiments were implemented to investigate the effects of circSCAF8 on the phenotype of PCa. We found that the knockdown of circSCAF8 in PCa cells suppressed the proliferation, migration, and invasion ability, while overexpression of circSCAF8 had the opposite effects. Similar results were observed in vivo. In a cohort of 85 patients who had undergone radical prostatectomy, circSCAF8 expression in PCa tissues was a powerful predictor of progression-free survival (HR = 2.14, *P* = 0.022). Mechanistically, circSCAF8 can function by binding to both miR-140-3p and miR-335 to regulate LIF expression and activate the LIF-STAT3 pathway that leads to the growth and metastasis of PCa. Collectively, our findings demonstrate that circSCAF8 contributes to PCa progression through the circSCAF8-miR-140-3p/miR-335-LIF pathway.

## Introduction

The clinical behavior of prostate cancer (PCa) is extremely heterogeneous. While some patients have potentially curable indolent cancer, others have incurable aggressive diseases leading to death [1]. Until now, several clinical and laboratory variables have been identified for the classification of PCa, including preoperative prostate-specific antigen (PSA) levels, Gleason score, and tumor stage [2–4]. However, it is well known that these methods are imperfect and limited because some patients within a given risk group have variable long-term outcomes [5]. Thus, additional biological markers that provide an objective evaluation of tumor biology are urgently needed to improve risk stratification for patients with PCa.

Although the genome of prostate tumors has been well investigated, circular RNAs (circRNAs) remain a poorly characterized component of the PCa transcriptome [6, 7]. Advances in high-throughput technologies might offer attractive and unbiased approaches for circular transcript biomarker development [8, 9]. The high stability and high conservation of circRNAs make them promising markers [6]. Recently, we and others [6, 7, 10] have applied next-generation sequencing to profile circRNAs as potential PCa biomarkers. Our work resulted in the analysis of a novel circRNA, circSCAF8 (Circbase ID: hsa_circ_0078367)—which is back-spliced of 11 exons (exons 2 to 12) of the SCAF8 gene (chr6:155095123–155131342), located at 6q25.2 amplicon—as an oncogenic factor associated with PCa progression.

CircRNAs belong to noncoding RNAs. Owing to the lack of 5′ caps and 3′ poly(A) tail, they possess special regulatory functions during biological processes [11]. Early studies suggested that circRNAs are a “splicing error” or a by-product of RNA processing. However, recent studies have shown the high prevalence of circRNAs in cancer [6]. Using more than 2000 clinical tumor samples across more than 40 cancer sites, a global analysis of circRNAs identified over 160,000 significantly expressed circRNAs [6]. Emerging evidence has shown that circRNAs can promote or inhibit oncogenesis in various kinds of cancer (e.g., hepatocellular carcinoma [12], breast cancer [13], glioblastoma [14], bladder cancer [15], renal cell carcinoma [16], and ovarian cancer [17]). Specifically for PCa, a total of 76,311 circRNAs have been identified [7]. Until now, several circRNAs have been reported to be associated with PCa progression [18–24]. For instance, circCSNK1G3 promotes PCa cell proliferation through the miR-181b/d/CBX7 signaling pathway [7]. CircFoxo3 suppresses the survival, migration, invasion, and chemoresistance to docetaxel of PCa cells by regulating the circFoxo3/Foxo3/EMT signals [25]. CircAMOTL1L accelerates PCa cell invasion and migration by sponging miR-193a-5p, upregulating vimentin, and downregulating E-cadherin, thereby resulting in epithelial-mesenchymal transition and PCa progression [19].

In our previous high-throughput RNA-sequencing studies of urine extracellular vesicles, we identified a novel circRNA referred to as circSCAF8, which was differentially expressed in a PCa group relative to a benign prostatic hyperplasia group and it was potentially relevant to tumorigenesis [10]. However, to the best of our knowledge, the function of circSCAF8 in PCa and the underlying mechanisms have not been thoroughly investigated.

In the present study, we first confirmed that the expression of circSCAF8 in urine extracellular vesicles was positively related to that in PCa tissues. Subsequently, in vitro and in vivo experiments showed that silencing of circSCAF8 suppressed the proliferation, invasion, and migration abilities of PCa cells. Mechanistically, circSCAF8 may function through sponging both miR-140-3p and miR-335, thereby activating the LIF-STAT3 pathway and facilitating PCa progression. Taken together, our results suggest that circSCAF8 may play a vital role in promoting PCa tumorigenesis and metastasis.

## Materials and methods

### Cell lines

All of the human PCa cell lines (PC3, 22RV1, LNCaP, C4-2, and DU-145), mouse prostate tumor cell line (RM-1), human prostate epithelial cell line (RWPE-1), and human embryonic kidney 293 T cell line were purchased from the American Type Culture Collection (ATCC, Manassas, VA). All of these cell lines were recently authenticated and tested for mycoplasma contamination. They were maintained at 37 °C with 5% CO_2_. The PC3, 22RV1, DU-145, and RM-1 cell lines were cultured in the RPMI 1640 medium (Bioss, Beijing, China). The C4-2, LNCaP, and 293 T cell lines were maintained in a DMEM medium (Biosharp, Guangzhou, China). The medium was supplemented with 10% fetal bovine serum (Gibco, South America). The RWPE-1 cell line was grown in K-SFM Basal medium, which contained both Keratinocyte-SFM (Gibco, Carlsbad, CA, USA) and BPE (Lonza, Basel, Switzerland).

### Plasmid construction and cell transfection

To knock down circSCAF8, two short-hairpin RNAs (shRNAs) specifically targeting the junction site of circSCAF8 were designed and synthesized by GenePharma (Suzhou, China). sh-NC vector was used as a negative control. pCDNA3.1-circSCAF8 was provided by GenePharma (Suzhou, China). For the overexpression of circSCAF8, the full-length cDNA of circSCAF8 was synthesized and cloned into the pCDNA3.1 vector by GenePharma (Suzhou, China). The mock vector without circSCAF8 sequence was used as a control. Then, the sh1-circSCAF8 vector, sh2-circSCAF8 vector, sh-NC vector, circSCAF8 vector, and mock vector were subcloned into lentivirus. To select stably transfected cells, lentiviruses carrying circSCAF8 vector, mock vector, sh1-circSCAF8 vector, sh2-circSCAF8 vector, and sh-NC vector were added into PC3 and 22RV1 cells as well as 1 µL Polybrene (5 µg/µL) (GenePharma, Suzhou, China). After 48 h, puromycin was added to the medium with a concentration of 5 µg/mL. After 72 h, the cells were no longer dead, and stably transfected cells were constructed for further research. The plasmids of LIF, si-LIF, and miR-140-3p/miR-335 mimics and inhibitors were designed and synthesized by RiboBio (Guangzhou, China). Then, the overexpression plasmids of LIF, si-LIF, and miR-140-3p/miR-335 mimics and inhibitors were transfected into PC3 and 22RV1 cells by Lipofectamine 3000 (Invitrogen, Carlsbad, CA, USA) in accordance with the manufacturer’s instructions. Quantitative real-time PCR (qRT-PCR) was used to verify the efficiency of overexpression and silencing. The sequences of the shRNAs and si-LIF were as follows:

sh1-circSCAF8: 5′-CTAAAACACTAAGTGTTGTAT-3′;

sh2-circSCAF8: 5′-CACTAAGTGTTGTATTCCCTG-3′;

sh-NC: 5′-TTCTCCGAACGTGTCACGT-3′; and

si-LIF: 5′-CAACAACCUGGACAAGCUAUGUGGC-3′.

### Luciferase reporter assay

The wild-type (WT) sequence or mutant (MUT) sequence of circSCAF8 or LIF 3′UTR, which corresponded to miR-140-3p and miR-335, were designed and synthesized by RiboBio (Guangzhou, China). Then, the sequences were inserted into the pmiR-Glo luciferase reporter vector (RiboBio, Guangzhou, China). Twenty-four hours before transfection, the 293 T cells (1 × 10^3^) were seeded into 96 wells. Then, the pmiR-Glo-circSCAF8-WT or pmiR-Glo-circSCAF8-MUT reporter plasmids, pmiR-Glo-LIF-WT or pmiR-Glo-LIF-MUT reporter plasmids, as well as miR-140-3p mimics, miR-335 mimics, and control miRNA were co-transfected into 293 T cells using Lipofectamine 3000 (Invitrogen, Carlsbad, CA, USA). The Firefly and Renilla luciferase activities were evaluated by a dual-luciferase reporter assay kit (Promega, Madison, WI, USA) after incubation for 48 h.

### RNA/gDNA extraction

Total RNA was extracted from PCa cell lines and tissues using TRIzol reagent (Invitrogen, Carlsbad, CA, USA) in accordance with the manufacturer’s protocol. The nuclear and cytoplasmic fractions were separated utilizing a Cytoplasmic & Nuclear RNA Purification Kit (Thorold, ON, Canada). RNA was reverse-transcribed into cDNAs with a PrimeScript RT Reagent Kit (Takara, Tokyo, Japan) following the manufacturer’s instructions. Genomic DNA (gDNA) was extracted from PC3 and 22RV1 cells in accordance with the PureLink Genomic DNA Mini Kit protocol (Thermo Fisher Scientific, MA, USA).

### RNase R treatment

The RNA extracted from the PC3 and 22RV1 cells was treated with RNase R (1.5 U/ug) (Geenseed, Guangzhou, China) for 30 min at 37 °C, and then the stability of circSCAF8 and its linear counterpart were measured by qRT-PCR.

### RNA pull-down assay by the specific biotin-coupled probe

The biotin-coupled circSCAF8 probe and oligo probe (RiboBio, Guangzhou, China) were performed to pull down the miRNAs sponged by circSCAF8. Approximately 1 × 10^7^ 22RV1 cells with circSCAF8 overexpression were lysed and mixed with the probes at room temperature for 16–24 h. Then, the streptavidin magnetic beads (MCE, Monmouth Junction, NJ, USA) were co-incubated with the lysis solution at room temperature for 2–4 h. After that, we set the lysis solution on a magnetic stand for 1 min and washed it with a washing buffer five times. Then, the magnetic beads were mixed with proteinase K (Sangon Biotech, Shanghai, China) and RNA PK buffer at 50 °C for 45 min, followed by 95 °C for 10 min to break the formaldehyde cross-links. The circSCAF8-miRNAs complex was purified by TRIzol reagent (Invitrogen, Carlsbad, CA, USA), and the abundance of miRNAs and circSCAF8 was estimated by qRT-PCR. The sequences of the circSCAF8 probe and oligo probe were as follows: circSCAF8 Probe 1: 5′-GGGAATACAACACTTAGTGT-3′;

circSCAF8 Probe 2: 5′-CAGGGAATACAACACTTAGT-3′; and

Oligo Probe: 5′-AGTCGGAACTGAAATCATGT -3′.

### Fluorescence in situ hybridization (FISH)

The location of circSCAF8 in PCa cells and the expression of circSCAF8 in PCa or benign prostatic hyperplasia tissues were determined by a FISH assay using the labeled-Cy3 of a circSCAF8 probe-FISH kit (RiboBio, Guangzhou, China). Images were photographed with a confocal fluorescence microscope (Leica, Wetzlar, Germany).

### qRT-PCR

In brief, the TB Green Premix Ex Taq (Takara, Tokyo, Japan) was utilized for qRT-PCR analysis. The reactions were conducted on a Roche LightCycler^®^ 480II PCR instrument (Basel, Switzerland). U6 or GAPDH was used as an internal standard control. The relative RNA expression levels were calculated by the 2^–ΔΔCT^ method. The primers used in the study are listed in Supplementary Table S1.

### Western blot analysis

Proteins were extracted from PCa cells using a Total Protein Extraction Kit (KeyGEN, Nanjing, China), and protein concentrations were measured by a BCA protein assay kit (KeyGEN, Nanjing, China). The detailed procedure was described in our previous study [26]. The antibodies against LIF (1:1000 dilution, ab138002), STAT3 (1:1000 dilution, ab68153), p-STAT3 (1:2000 dilution, ab267373), Ago2(1:1000 dilution, ab186733) and CyclinD1 (1:1000 dilution, ab226977) were obtained from Abcam (Burlingame, CA, USA); and the antibodies against VEGFA (1:1000 dilution, 65373), MMP-2 (1:1000 dilution, 4022), MMP-9 (1:1000 dilution, 2270), and GAPDH (1:1000 dilution, 3683) were acquired from Cell Signaling Technology (Beverly, MA, USA). The secondary antibody (1:5000 dilution, bs-0295G) was provided by Bioss (Beijing, China). Chemiluminescent signals were detected using Western ECL Substrate (Advansta, Menlo Park, CA, USA). Images were taken using a ChemiDoc Imaging System (Bio-Rad, Hercules, CA, USA).

### Immunohistochemistry (IHC)

The murine tumor paraffin sections were deparaffinized and dehydrated by xylene and a series of graded ethanol solutions. Subsequently, the paraffin sections were incubated with the antibodies against LIF (1:1000 dilution; Abcam, ab138002), p-STAT3 (1:100 dilution; Abcam, ab267373), CyclinD1 (1:250 dilution; Abcam, ab226977), VEGFA (1:100 dilution; Cell Signaling Technology, 65373), MMP-2 (1:200 dilution; Cell Signaling Technology, 4022), and MMP-9 (1:500 dilution; Cell Signaling Technology, 2270) at 4 °C overnight. Then, the paraffin sections were incubated with the secondary antibody (1:500 dilution; bs-0295G, Bioss, Beijing, China) for 1 h at room temperature. After washing with phosphate-buffered saline (PBS) three times, the paraffin sections were stained with diaminobenzidine and hematoxylin and then treated with a series of graded ethanol solutions and xylene. Finally, neutral balata was used to fix the sections, and they were photographed with a fluorescence microscope (Olympus, Tokyo, Japan). The IHC score was measured as follows: (1) staining area score: 0, <5%; 1, 5–25%; 2, 25–50%; 3, 50–75%; and 4, > 70%; (2) staining intensity score: 0, no staining; 1, weak staining; 2, moderate staining; and 3, intense staining; and (3) total staining score was estimated through staining intensity and staining area. Samples with a score ≥6 were considered to indicate high expression, while those with a score <6 were considered to have low expression [27].

### Cell proliferation assay

CCK-8, colony formation, and 5-ethynyl2′deoxyuridine (EdU) assays were used to measure the proliferation of PCa cells. For the CCK-8 assay, 2000 cells were seeded in a 96-well plate and conventionally cultured for 24, 48, and 72 h. Then, 10 µL of CCK-8 reagent (Dojindo, Kumamoto, Japan) was added to each well for 2.5 h at 37 °C. The absorbance in each well was recorded by a microplate reader (Bio-Rad, USA). For the colony formation assay, 500 cells were seeded in a six-well plate and grown for 3 weeks. Then, the cells were stained with 0.1% crystal violet for 15 min and washed with PBS two times. Photographs were taken, and the number of colonies was counted. For the EDU assay, 5000 cells were seeded in a 48-well plate with a climbing slice. After 24 h, EDU reagents (BD Bioscience, CA, USA) were added to each well following the manufacturer’s instructions. Images were taken with a fluorescence microscope (Olympus, Tokyo, Japan).

### Transwell invasion and migration assays

Transwell invasion and migration assays were performed in accordance with our previous report [26].

### Wound healing assay

In brief, 5 × 10^5^ PCa cells were seeded in a six-well plate and grown to 70% confluence, and the wound was created in the cell layer by a white pipette tip. At the time points of 0 and 24 h, photographs were taken by a microscope system (Olympus, Tokyo, Japan) and the migrated distance was calculated.

### Cell cycle and apoptosis assays

For the cell cycle assay, PCa cells were trypsinized, washed twice with PBS, and fixed in precooled 80% ethanol at 4 °C overnight. After ethanol fixation, the cells were washed twice with PBS, followed by incubation with 50 µg/mL RNase A (KeyGEN, Nanjing, China) and 10 µg/mL propidium iodide (KeyGEN, Nanjing, China) at 37 °C for 15 min. Then, the cell cycle was analyzed by flow cytometry (Becton Dickinson FACS Calibur, NY, USA). For the cell apoptosis assay, PCa cells were washed twice with precooled PBS and then trypsinized without EDTA. Subsequently, the cells were stained with Annexin V-FITC and PI (KeyGEN, Nanjing, China) at room temperature for 5 min in the dark, and the ratio of apoptotic cells was determined by flow cytometry (Becton Dickinson FACS Calibur, NY, USA).

### Animal study

All of the animal experiments were approved by the Animal Ethics Committee of the Third Affiliated Hospital of Sun Yat-Sen University.

A subcutaneous tumor model and an orthotopic tumor model were used to construct the tumor formation assay in BALB/c nude mice. For the subcutaneous model, 4–6-week-old male BALB/c nude mice were randomly divided into the sh1-circSCAF8 group (*n* = 8) and the sh-NC group (*n* = 8). Approximately 2 × 10^6^ PC3 cells were resuspended in the medium containing 20% Matrigel (BD, San Jose, CA, USA), and then subcutaneously injected into the right flank of each mouse. Tumor growth was measured every week, and tumor volume was calculated according to the formula (length × width^2^/2). All of the mice were euthanized after 4 weeks, and the tumor weight was determined by electronic balance.

For the orthotopic model, 4–6-week-old male BALB/c nude mice were randomly divided into the sh1-circSCAF8 group (*n* = 7) and the sh-NC group (*n* = 7). Then, they were anesthetized, and ~2 × 10^6^ PC3 cells were mixed in the medium containing 20% Matrigel (BD, San Jose, CA, USA) and then injected into the ventral prostate. In vivo imaging was performed every week to observe orthotopic tumor formation using an AniView100 system (BLT, Guangzhou, China). After 5 weeks, all of the mice were euthanized, and the primary tumors in the prostate were removed and weighed. The tissue sample sections were prepared as previously described [26, 28], and histopathological evaluation was conducted on hematoxylin and eosin (H&E)–stained paraffin sections of each sample. As mentioned above, IHC staining was performed on paraffin sections at the Third Affiliated Hospital of Sun Yat-Sen University.

For the metastasis model, 4–6-week-old male NOD/SCID mice were randomly divided into the sh-NC group (*n* = 7), sh1-circSCAF8 group (*n* = 7), sh1-circSCAF8+inh-NC group (*n* = 7), sh1-circSCAF8+inh-140-3p group (*n* = 7), sh1-circSCAF8+inh-335 group (*n* = 7), and sh1-circSCAF8+inh-140-3p/335 group (*n* = 7). Approximately 2 × 10^6^ RM-1 cells were resuspended in the medium and then injected into the tail vein of each mouse. In vivo imaging was performed every week to observe the formation of metastases using an AniView100 system (BLT, Guangzhou, China). After 4 weeks, all of the mice were euthanized, and the freshly dissected livers, lungs, kidneys, spleens, and macroscopic metastases were examined and photographed. The tissue samples were prepared and stained with H&E as mentioned above.

### Study on patients’ tissues

A total of 85 PCa tissues and 30 benign prostatic hyperplasia tissues were obtained from the Third Affiliated Hospital of Sun Yat-Sen University. These 85 patients with PCa underwent radical prostatectomy, and the 30 patients with benign prostatic hyperplasia underwent transurethral prostatectomy. In addition, we collected 30 preoperative first-catch non-digital rectal examination urine samples from 30 of the 85 PCa patients and 30 preoperative first-catch non-digital rectal examination urine samples from the 30 patients with benign prostatic hyperplasia. The urine sample preparation, extracellular vesicle isolation, and characterization were previously described [10]. This study was approved by the Ethical Committee of the Third Affiliated Hospital of Sun Yat-Sen University. Written informed consent was obtained from all of the patients.

### Bioinformatics analysis

Circinteractome database (https://circinteractome.nia.nih.gov/) was used to predict the possible miRNAs that could bind to circSCAF8. miRanda (http://www.microrna.org) and Targetscan (http://www.targetscan.org) were used to predict the target genes of miR-140-3p and miR-335.

### Statistical analysis

All of the statistical analyses were performed by SPSS 20.0 (SPSS, Chicago, IL, USA), R software (version 3.6.1), and GraphPad Prism 8.0 (GraphPad Software Inc., CA, USA). Student’s *t*-test, one-way ANOVA, or chi-square test was used to analyze the differences between groups. Kaplan–Meier curves and log-rank test were used to evaluate progression-free survival. The Cox proportional hazards regression model was constructed to estimate the effect of circSCAF8 on progression-free survival. The correlations between the groups were assessed using Pearson correlation analysis. A two-tailed *P* value lower than 0.05 was considered to be statistically significant.

## Results

### CircSCAF8 is positively associated with PCa progression

**Fig. 1:**
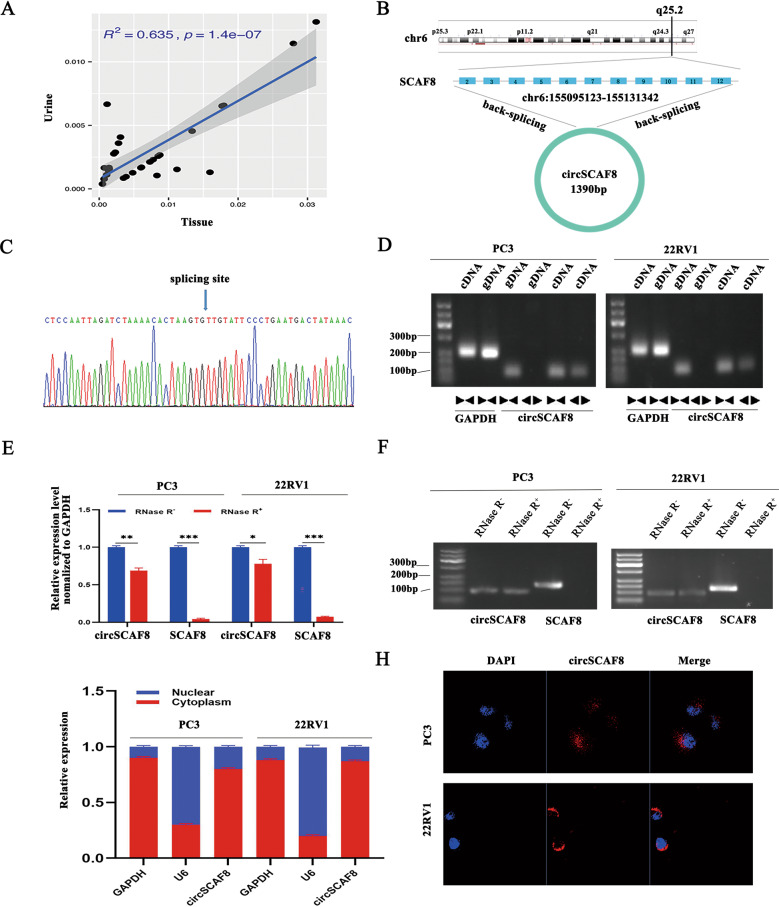
The validation and characterization of circSCAF8 in PCa. **A** The Spearman correlation analysis of circSCAF8 level between the tumor tissue and matched urine extracellular vesicles in 30 PCa patients**. B** Schematic illustration showing circSCAF8 derived from exons 2 to 12 of SCAF8. **C** Rolling circle reverse transcription and Sanger sequencing were used to confirm the full length of circSCAF8 in PC3 cells. The arrow indicated the special back splicing junction site of circSCAF8. **D** Divergent and convergent primers were used to detect circular RNAs in cDNA and gDNA, divergent primers can detect the existence of circular RNAs in cDNA but not in gDNA. GAPDH was used as a control for a linear RNA transcript. **E**, **F** The abundances of circSCAF8 and SCAF8 mRNA were determined by PCR and qRT-PCR after treatment with RNase R in PC3 and 22RV1 cells. **G**, **H** Cytoplasmic-nuclear fractionation assay and FISH showed that circSCAF8 was mainly localized in the cytoplasm of PC3 and 22RV1 cells. GAPDH and U6 were used as cytoplasm control and nuclear control, respectively. Error bars represent the standard deviation (SD) of three independent experiments. ^*^P < 0.05; ^**^P < 0.01; ^***^P < 0.001. PCa prostate cancer, FISH fluorescence in situ hybridization.

Previously, we used high-throughput RNA sequencing to identify candidate urine extracellular vesicle circRNAs that were increased in patients with high-grade PCa [10]. In total, we identified 2231 urine extracellular vesicle circRNAs that had significantly different levels between individuals with benign prostatic hyperplasia (*n* = 11) and those with high-grade PCa (*n* = 11). Of these 2231 circRNAs, 18 circRNAs were upregulated and 2213 were downregulated in high-grade PCa. Because we were mainly interested in the potential circRNAs that are positively associated with PCa progression, we focused on the 18 circRNAs that were increased in patients with high-grade PCa. Then, we conducted the Spearman correlation analysis of the levels of the 18 circRNAs between the tumor tissue and the matched urine extracellular vesicles in 30 PCa patients. The correlation analysis showed that hsa_circ_0078367 had the highest *R*^*2*^ (0.635). Thus, we chose hsa_circ_0078367 for further study (Fig. 1A and Supplementary Fig. 1).

Given that hsa_circ_0078367 is derived from SCAF8, we termed it circSCAF8. CircSCAF8 is back-spliced of 11 exons (exons 2 to 12) of the SCAF8 gene (chr6:155095123–155131342), located at 6q25.2 amplicon (Fig. 1B). The rolling circle reverse transcription and Sanger sequencing were performed to confirm the entire sequence of circSCAF8 in PC3 cells (Fig. 1C and Supplementary Fig. S2A–D). To confirm the formation of circSCAF8, circSCAF8 circular transcripts and SCAF8 linear transcripts were amplified by divergent and convergent primers, respectively. The PCR results showed that circSCAF8 was amplified from both primers in cDNA, while only convergent primers detected the existence of circSCAF8 in genomic DNA (gDNA) (Fig. 1D). In addition, circSCAF8 was more resistant to RNase R digestion than linear SCAF8 (Fig. 1E, F). The localization of circSCAF8 in PC3 and 22RV1 cells was evaluated via cytoplasmic-nuclear fractionation assay and FISH, showing that circSCAF8 was principally enriched in the cytoplasm (Fig. 1G, H). Collectively, these results confirm that circSCAF8 possesses a circular structure and is more stable than its linear counterpart.

### CircSCAF8 is highly expressed in PCa tissue and urine extracellular vesicles

**Fig. 2:**
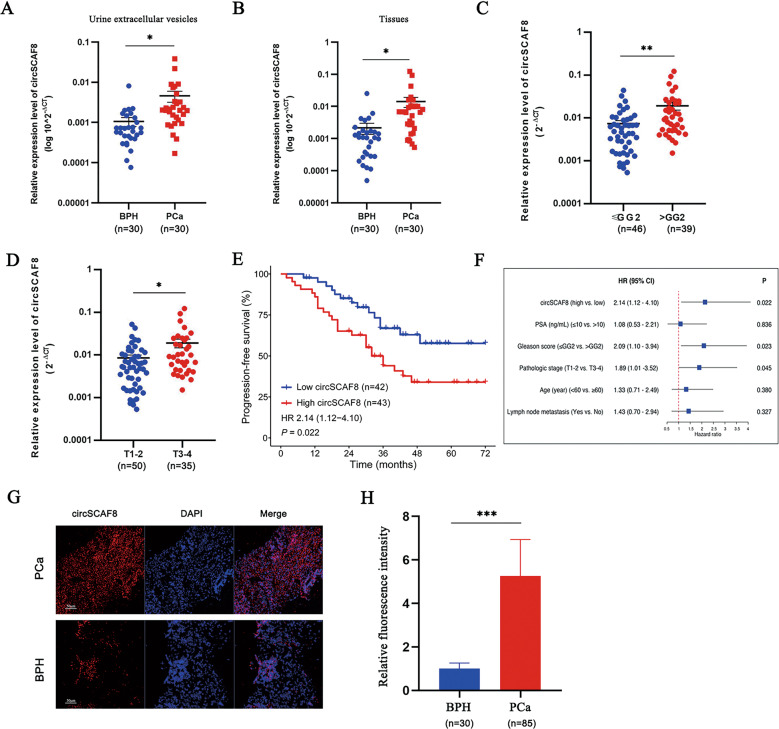
circSCAF8 is upregulated in PCa tissues and urine extracellular vesicles. **A**, **B** The expression profile of circSCAF8 in urine extracellular vesicles (**A**) and tissues (**B**) from patients with PCa (*n* = 30) and those with benign prostatic hyperplasia (*n* = 30). **C**, **D** The circSCAF8 expression levels (stratified by grade and stage) in PCa tissues from a cohort (*n* = 85) who underwent radical prostatectomy. **E** Kaplan–Meier curves of progression-free survival according to the circSCAF8 expression level in PCa tissues (*n* = 85). **F** Cox regression analysis of the circSCAF8 expression level with progression-free survival. **G**, **H** Representative images and quantitative analyses for the circSCAF8 expression in PCa/benign prostatic hyperplasia tissue via FISH. ^*^P < 0.05; ^**^P < 0.01; ^***^P < 0.001. PCa prostate cancer, FISH fluorescence in situ hybridization, GG grade group.

To validate the expression profile of circSCAF8 in PCa tissues and urine extracellular vesicles, qRT-PCR was employed in 30 PCa/benign prostatic hyperplasia tissue samples and urine extracellular vesicles. Compared with urine extracellular vesicles and tissue samples from benign prostatic hyperplasia patients, circSCAF8 has significantly upregulated in urine extracellular vesicles and tissue samples from PCa patients (Fig. 2A, B). Similar results were obtained using FISH in PCa/benign prostatic hyperplasia tissue samples (Fig. 2G, H). Furthermore, the correlation analysis showed that circSCAF8 expression in urine extracellular vesicles significantly and positively correlated with the matched PCa tissues (Fig. 1A). In addition, we evaluated the prognostic ability of circSCAF8 expression in PCa tissues from the cohort of patients (*n* = 85) who had undergone radical prostatectomy. Patients with high grade (> Grade Group [GG]2) or stage (T3–4) had higher circSCAF8 expression than patients with low grade (≤GG2) or stage (T1–2) (Fig. 2C, D and Supplementary Table S2). Using the Cox proportional hazards regression model, circSCAF8 expression remained a powerful predictor of radiographic progression-free survival (HR = 2.14, 95% CI, 1.12–4.10, *P* = 0.022) (Fig. 2E, F). Radiographic progression was defined as ≥20% increase in the sum of the diameters of soft-tissue lesions evaluated by MRI or CT using Response Evaluation Criteria in Solid Tumors [29] or at least two new bone lesions on technetium-99 bone scanning.

### CircSCAF8 promotes the proliferation, invasion, and migration of PCa cells

**Fig. 3:**
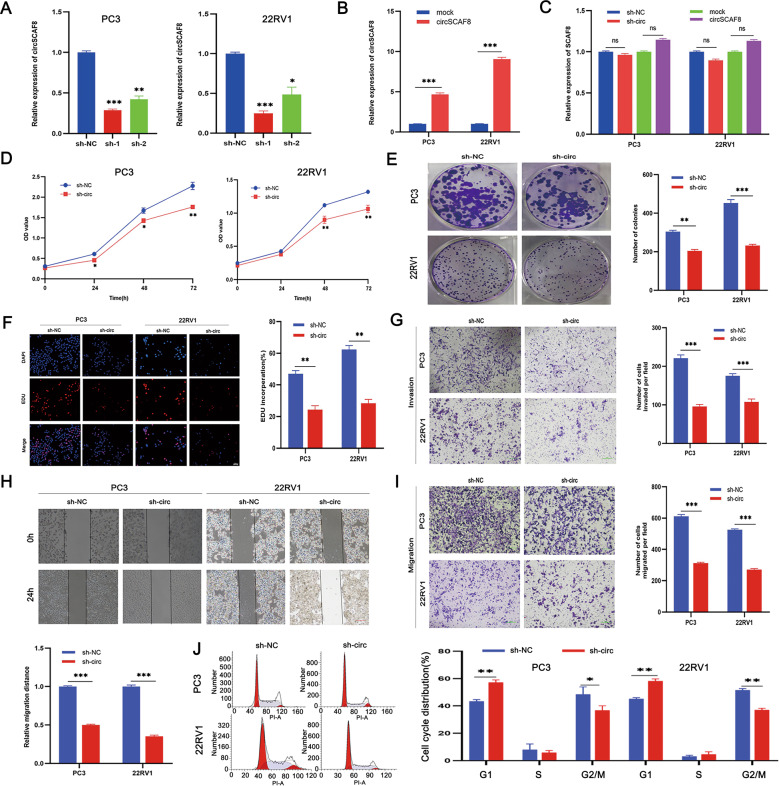
Knockdown of circSCAF8 can suppress the ability of proliferation, invasion, and migration of PCa cells. **A** The knockdown efficiency of the two shRNAs that target circSCAF8 in PC3 and 22RV1 cells. **B** The expression level of circSCAF8 after transfected with circSCAF8 expression vectors in PC3 and 22RV1 cells. **C** qRT-PCR analysis of SCAF8 mRNA expression after treatment with sh1-circSCAF8 and circSCAF8 expression vectors. **D**–**F** The proliferation ability of PC3 and 22RV1 cells after knockdown of circSCAF8 was measured by CCK-8 **D**, colony formation (**E**), and EDU **F** assays. **G** The invasion ability of PC3 and 22RV1 cells was determined using a transwell invasion assay. **H**, **I** Transwell migration and wound healing assays were implemented to assess the migration ability of PC3 and 22RV1 cells after the knockdown of circSCAF8. **J** Cell cycle analysis revealed that PCa cells were blocked at the G1 phase after knockdown of circSCAF8. Error bars represent the SD of three independent experiments. ^*^P < 0.05; ^**^P < 0.01^; ***^P < 0.001; ns nonsignificant, PCa prostate cancer, EDU 5-ethynyl2′deoxyuridine.

Analysis of endogenous circSCAF8 expression across a panel of five PCa cell lines by means of the qRT-PCR assay showed that their expression of circSCAF8 was higher compared with that of one human prostate epithelial cell line (RWPE-1) (Supplementary Fig. 3). To clarify the functional role of circSCAF8 in PCa cells, we designed two shRNAs specifically targeting the back-splice junction sites of circSCAF8. Meanwhile, the full length of circSCAF8 was cloned into expression vectors to construct the overexpression vector. qRT-PCR was applied to validate the efficiency of overexpression or inhibition of circSCAF8 in PC3 and 22RV1 cells. These results showed that both shRNAs were able to stably inhibit the expression of circSCAF8; however, sh1-circSCAF8 had excellent knockdown efficiency and was selected for further investigation (Fig. 3A, B). Notably, the mRNA expression of the linear counterparts of circSCAF8 was not influenced by the upregulated or downregulated circSCAF8 (Fig. 3C). Subsequently, the proliferation capacity of PC3 and 22RV1 cells was measured by CCK-8, colony formation, and EdU assays. The results showed that silencing of circSCAF8 greatly suppressed the proliferation ability of PC3 and 22RV1 cells, while overexpression of circSCAF8 accelerated cell proliferation (Fig. 3D–F and Supplementary Fig. 4A–C). Meanwhile, the invasive and migratory capabilities of PC3 and 22RV1 cells were investigated by transwell and wound healing assays. The results indicated that the invasion and migration abilities were significantly inhibited by the knockdown of circSCAF8 (Fig. 3G–I), while overexpression of circSCAF8 showed the opposite effect (Supplementary Fig. 4D–F). Finally, cell cycle and apoptosis assays were performed to determine the role of circSCAF8 in PC3 and 22RV1 cells. The cell cycle analysis revealed that PC3 and 22RV1 cells were blocked at the G1 phase after knockdown of circSCAF8 (Fig. 3J), while flow cytometry analysis showed that there was no significant impact on cell apoptosis when compared with the control vector (Supplementary Fig. 4G). Taken together, these function experiments suggest that circSCAF8 has an oncogenic role in PCa cells.

### CircSCAF8 functions through sponging miR-140-3p and miR-335

**Fig. 4:**
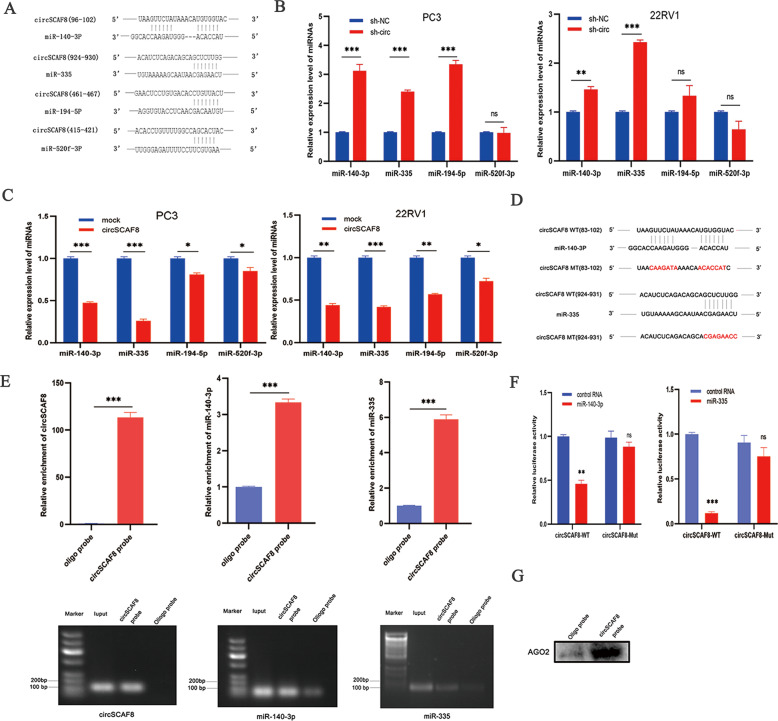
circSCAF8 functions through sponging both miR-140-3p and miR-335 in PCa cells. **A** Bioinformatic analyses revealed that circSCAF8 could sponge four miRNAs (miR-140-3p, miR-335, miR-520f-3p, and miR-194-5p) with relatively high scores. **B**, **C** The expression level of four prediction miRNAs (miR-140-3p, miR-335, miR-520f-3p, and miR-194-5p) was analyzed by qRT-PCR after knockdown or overexpression of circSCAF8 in PC3 and 22RV1 cells. **D**, **F** The dual-luciferase reporter assay showed that circSCAF8 can sponge both miR-140-3p and miR-335, and can suppress the luciferase ability. **E** RNA pull-down through biotin-yoked circSCAF8 probe was executed in 22RV1 cells with circSCAF8 overexpression, then RT-PCR and qRT-PCR were applied to verify the enrichment of circSCAF8, miR-140-3p, and miR-335. **G** Western blot analysis showed that AGO2 protein was abundantly enriched in the circSCAF8 probe through RNA pull-down assay. Error bars represent the SD of three independent experiments. ^*^P < 0.05; ^**^P < 0.01; ^***^P < 0.001; ns nonsignificant, PCa prostate cancer.

According to the theory that circRNAs can function as miRNA sponges [30], we hypothesized that circSCFA8 may capture miRNAs by competitive endogenous RNA (ceRNA) mechanisms. To verify this assumption, bioinformatic analysis was first implemented to predict miRNAs that can bind to circSCAF8 through the Circinteractome database (https://circinteractome.nia.nih.gov/). Four miRNAs (miR-140-3p, miR-520f-3p, miR-194-5p, and miR-335) had relatively high scores (Fig. 4A). Next, we investigated the influence of circSCAF8 on the four miRNAs. These results showed that miR-335 and miR-140-3p were greatly changed in PC3 and 22RV1 cells after overexpression or downregulation of circSCAF8 (Fig. 4B, C). We then hypothesized that miR-335 and miR-140-3p may directly bind to circSCAF8. Subsequently, dual-luciferase reporter assays were executed to confirm this hypothesis. The reporter plasmids of circSCAF8 with WT and MUT were constructed (Fig. 4D) and then co-transfected with miR-140-3p/miR-335 mimics and control RNAs, respectively. We found that miR-140-3p mimics and miR-335 mimics significantly inhibited cirSCAF8-WT luciferase activity, while the luciferase activity of MUT changed insignificantly (Fig. 4F). Given the concept that AGO2 protein participates in the process of many miRNAs by forming the RNA-AGO2 complex [31, 32], RNA pull-down through biotin-coupled circSCAF8 probe was performed to authenticate the relationship between circSCAF8, miR-140-3p, and miR-335, and to ascertain the role of AGO2 protein in the progression. The results showed that miR-140-3p, miR-335, and AGO2 protein were abundantly enriched in the circSCAF8 probe rather than in the control probe in 22RV1 cells with overexpression of circSCAF8 (Fig. 4E, G). Thus, these data suggest that circSCAF8 may function through sponging both miR-140-3p and miR-335.

### The tumor-promoting effects caused by circSCAF8 can be partially reversed by miR-140-3p and miR-335

**Fig. 5:**
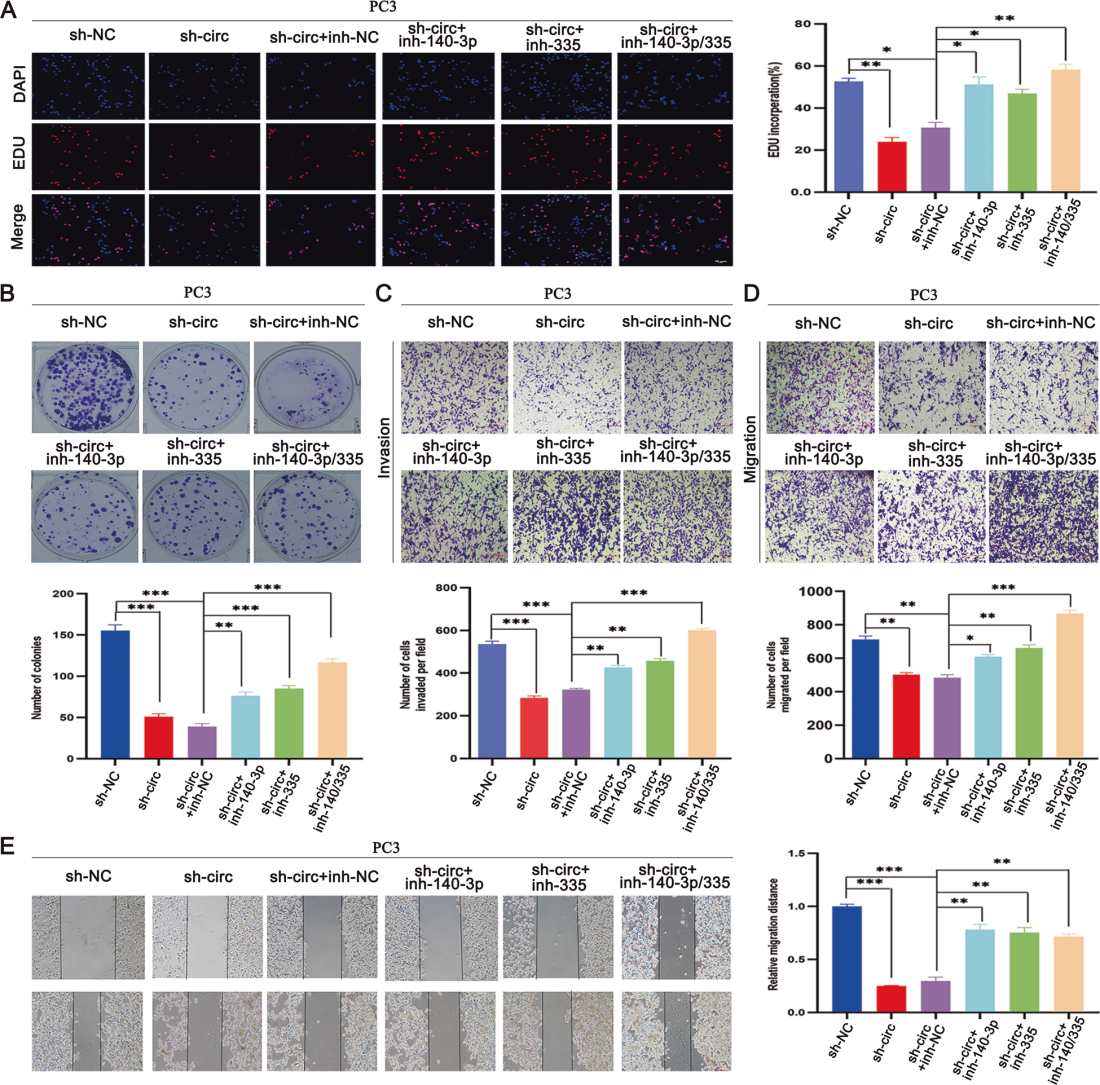
miR-140-3p and miR-335 can partially rescue the tumor-suppression effects caused by the knockdown of circSCAF8 in PCa cells. **A**, **B** EDU and colony formation assays were carried out to detect the proliferation ability of PC3 cells in the circSCAF8 knockdown group after being transfected with miR-140-3p inhibitors, miR-335 inhibitors, and miR-140-3p/miR-335 inhibitors, respectively. **C**–**E** The invasion and migration abilities of PC3 cells with circSCAF8 knockdown treated with miR-140-3p inhibitors, miR-335 inhibitors, and miR-140-3p/miR-335 inhibitors were measured by transwell invasion assay **C**, transwell migration assay (**D**), and wound healing assay (**E**). Error bars represent the SD of three independent experiments. ^*^P < 0.05; ^**^P < 0.01; ^***^P < 0.001. PCa prostate cancer, EDU 5-ethynyl2′deoxyuridine.

To investigate the relationships among circSCAF8, miR-140-3p, and miR-335, rescue experiments of co-transfecting sh-circSCAF8 and miRNA inhibitors or circSCAF8 vector and miRNA mimics were performed in PC3 and 22RV1 cells. As shown by EdU, wound healing, transwell, and colony formation assays, ectopic expression of miR-140-3p and miR-335 significantly suppressed the invasion, migration, and proliferation abilities caused by overexpression of circSCAF8 in PC3 and 22RV1 cells, while miR-140-3p and miR-335 inhibitors eliminated the suppression effects caused by circSCAF8 knockdown in PC3 and 22RV1 cells. More importantly, the incorporation of two miRNA mimics or inhibitors displayed a greater rescue effect (Fig. 5A–E and Supplementary Fig. 5A–E). Taken together, these data further confirm the ceRNA mechanism for circSCAF8 in PCa.

### LIF is the direct target gene of both miR-140-3p and miR-335

**Fig. 6:**
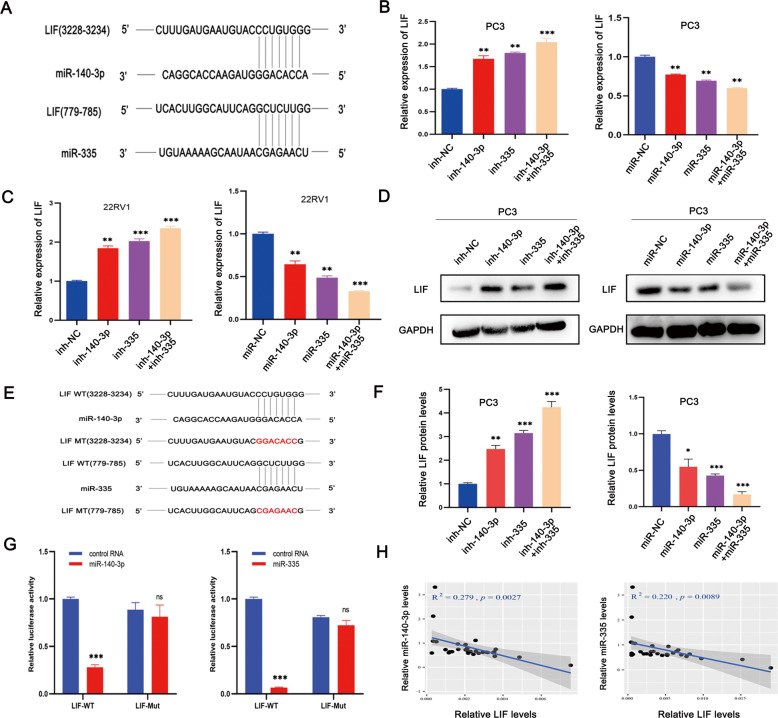
LIF is the direct target gene of both miR-140-3p and miR-335. **A** Bioinformatic analysis predicted that LIF was the potential direct target gene of both miR-140-3p and miR-335. **B**, **C** qRT-PCR analysis showed that miRNA inhibitors increased the expression level of LIF, while miRNA mimics suppressed the expression level of LIF. **D**, **F** Western blot results showed the expression level of LIF after transfecting with miRNA inhibitors or miRNA mimics in PC3 cells. **E**, **G** Dual-luciferase reporter assays revealed that both miR-140-3p and miR-335 could bind to the 3′ UTR of LIF, and could suppress the luciferase ability. **H** Pearson correlation analyses showed that both miR-140-3p and miR-335 in PCa tissues (*n* = 30) were negatively correlated with LIF. Error bars represent the SD of three independent experiments. ^**^P < 0.01; ^***^P < 0.001; ns nonsignificant.

According to the theory that miRNAs can target the 3′UTR of mRNA to regulate the expression of the target mRNA [33], bioinformatics analyses were conducted using miRanda (http://www.microrna.org) and Targetscan (http://www.targetscan.org) to identify the potential target genes of miR-140-3p and miR-335. The data showed that LIF was the direct target gene of both miR-140-3p and miR-335 (Fig. 6A). Subsequently, the expression levels of LIF in PCa cells after transfecting miRNA mimics or miRNA inhibitors were assessed by qRT-PCR and western blot. These results demonstrated that both miRNA mimics suppressed the expression level of LIF, while miRNA inhibitors increased the expression level of LIF (Fig. 6B, C, D, F). In addition, we evaluated the abundance of miR-140-3p, miR-335, and LIF in PCa tissues (n = 30) and found that both miR-140-3p and miR-335 negatively correlated with LIF (Fig. 6H). Furthermore, WT and MUT dual-luciferase reporter plasmids were synthesized. Luciferase reporter analyses revealed that co-transfection of LIF-WT plasmids and miRNA mimics evidently reduced the luciferase activity, while the luciferase activity did not change remarkably in the case of co-transfection of LIF-MUT plasmid and miRNA mimics (Fig. 6E, G). These data show that LIF is the direct target gene of both miR-140-3p and miR-335.

### CircSCAF8 promotes PCa progression via the circSCAF8-miR-140-3p/miR-335-LIF axis

**Fig. 7:**
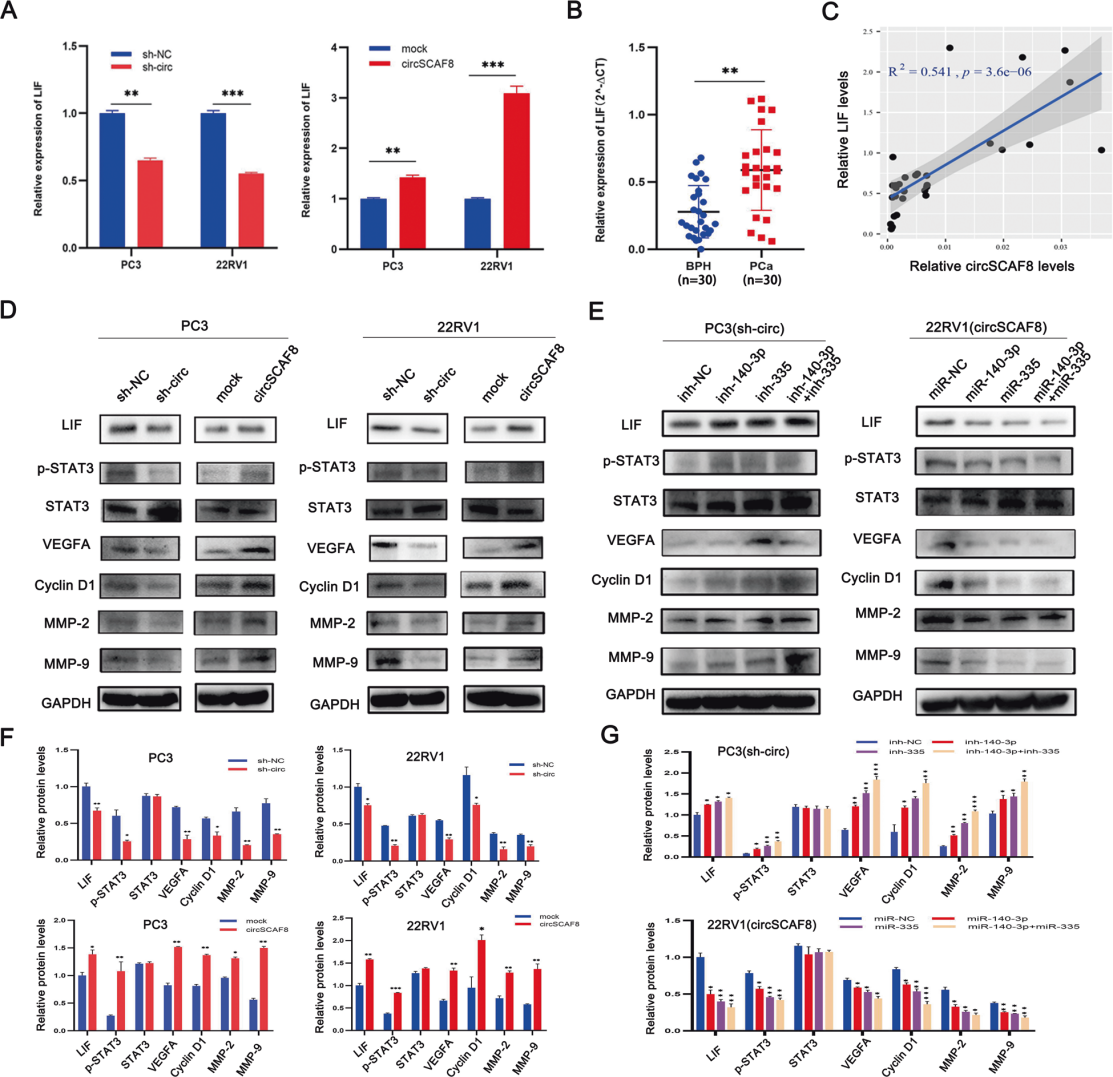
CircSCAF8 promotes PCa progression via circSCAF8-miR-140-3p/miR-335-LIF axis. **A** The relative expression of LIF was measured by qRT-PCR after knockdown or overexpression of circSCAF8 in PC3 and 22RV1 cells. **B** The relative expression of LIF was detected in 30 PCa tissues and 30 benign prostatic hyperplasia tissues by qRT-PCR. **C** Pearson correlation analyses showed that the expression level of circSCAF8 was positively correlated with that of LIF. **D**, **F** Knockdown of circSCAF8 lead to the decrease of LIF and its downstream signaling pathway proteins (p-STAT3, MMP-2, MMP-9, VEGFA, and CyclinD1), while overexpression of circSCAF8 had an opposite effect. **E**, **G** miR-140-3p/miR-335 inhibitors or mimics could partially reverse the effects of circSCAF8 on LIF and its downstream signaling pathway proteins (p-STAT3, MMP-2, MMP-9, VEGFA, and CyclinD1). Error bars represent the SD of three independent experiments. ^**^P < 0.01; ^***^P < 0.001. PCa prostate cancer.

**Fig. 8:**
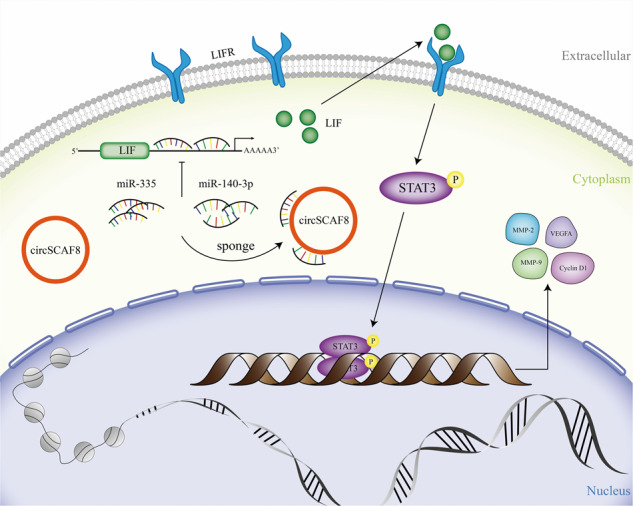
A schematic model for the circSCAF8-miR-140-3p/miR-335-LIF pathway. A schematic model showing the mechanism underlying circSCAF8 as a ceRNA for miR-140-3p and miR-335. ceRNA competitive endogenous RNA.

To explore the underlying mechanisms by which circSCAF8 regulates PCa progression, we first evaluated the expression level of LIF by silencing or overexpressing circSCAF8 in PC3 and 22RV1 cells. We found that the downregulation of circSCAF8 decreased the expression level of LIF, while the upregulation of circSCAF8 increased the expression level of LIF (Fig. 7A). However, the expression level of circSCAF8 was not influenced by the upregulation or downregulation of LIF (Supplementary Fig. 6A). Second, the expression levels of LIF in PCa (*n* = 30) and benign prostatic hyperplasia tissue samples (*n* = 30) were determined by qRT-PCR. As expected, the expression level of LIF in PCa tissues was significantly increased compared with that in benign prostatic hyperplasia tissues (Fig. 7B). Furthermore, the expression level of LIF positively correlated with the expression level of circSCAF8 (Fig. 7C). Subsequently, rescue experiments were designed to test the roles of miR-140-3p and miR-335 in the regulation of LIF caused by circSCAF8. The results suggested that the upregulation or downregulation of LIF caused by circSCAF8 overexpression or downregulation could be partially reversed by miRNA mimics or miRNA inhibitors, respectively (Fig. 7E, G). In addition, we found that knockdown of circSCAF8 led to a decrease in LIF, p-STAT3, MMP-2, MMP-9, VEGFA, and CyclinD1, while overexpression of circSCAF8 had an opposite effect (Fig. 7D, F). Similarly, miRNA mimics or inhibitors partially reversed these effects (Fig. 7E, G). In addition, the phenotypic effects triggered by the silencing of circSCAF8 in PC3 cells were dampened by LIF overexpression as evidenced by EdU, transwell, and colony formation assays (Supplementary Fig. 6B–E). In 22RV1 cells, LIF inhibition could phenocopy the silencing of circSCAF8, as reflected in EdU, transwell, and colony formation assays (Supplementary Fig. 6F–H). Collectively, we confirmed that circSCAF8 promotes PCa progression through the circSCAF8-miR-140-3p/335-LIF axis (Fig. 8).

### CircSCAF8 promotes the growth and metastasis of PCa cells in vivo

**Fig. 9:**
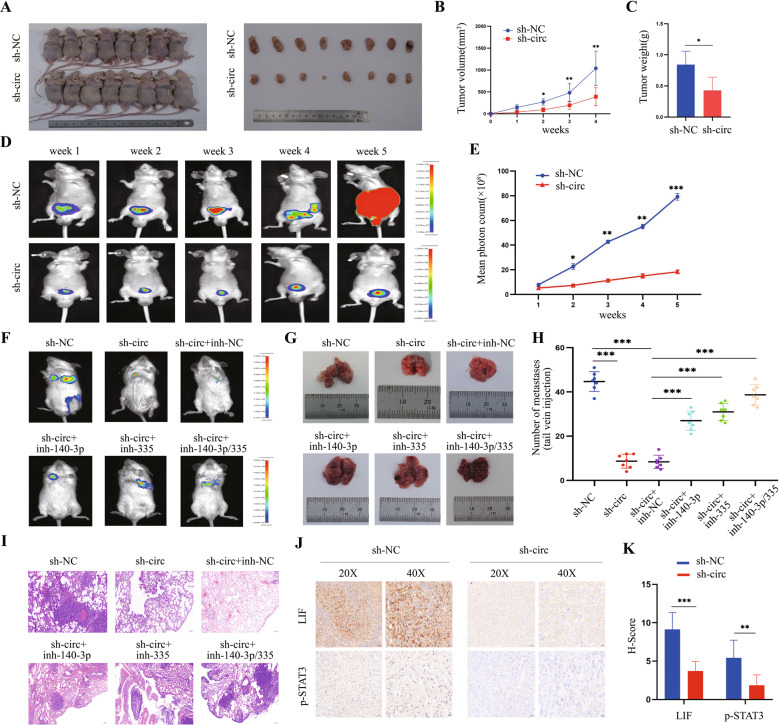
CircSCAF8 promotes growth and metastasis of PCa cells in vivo. **A** BALB/c nude mice were anesthetized and sacrificed at the experimental endpoint, and subcutaneous xenograft tumors formed by PC3 cells were dissected and photographed (*n* = 8 for each group). **B** The subcutaneous tumor volumes were measured once a week and the growth curves were shown. **C** Each subcutaneous tumor at the experimental endpoint was weighed. **D** Orthotopic tumor growth of PC3 cells was monitored weekly by in vivo bioluminescence imaging. Two representative images are shown for each time point. (*n* = 7 for each group) **E** Orthotopic tumor bioluminescence signals were measured by mean photon radiance. **F**–**I** Representative images **F**, **G**, hematoxylin and eosin staining (**I**), and quantification (**H**) of lung metastatic colonization of NOD/SCID mice treated with a tail-vein injection of RM-1 cells (*n* = 7 for each group). **J**, **K** Representative images of IHC staining and quantitative analyses showing the intratumoral expression of LIF and p-STAT3 within orthotopic xenografts (*n* = 7 for each group). Error bars represent the SD. ^*^P < 0.05; ^**^P < 0.01; ^***^P < 0.001. PCa prostate cancer, IHC immunohistochemistry.

To evaluate the role of circSCAF8 in vivo, we established a tumor metastasis model and subcutaneous and orthotopic xenograft tumor models in mice. In the subcutaneous xenograft model, the tumor volume and weight were remarkably lower in the sh1-circSCAF8 group than in the control group (Fig. 9A–C). In the orthotopic xenograft model, compared with the control group, tumor bioluminescence signals in the sh1-circSCAF8 group were weaker for each time point (Fig. 9D, E). Moreover, in NOD/SCID mice metastasis models, we found that knockdown of circSCAF8 significantly suppressed spontaneous lung metastases, while miR-140-3p inhibitors and miR-335 inhibitors partially reversed the effects (Fig. 9F–I). Subsequently, the expression of LIF and its downstream proteins in orthotopic tumors was determined by IHC, and the results showed that the intratumoral expression levels of LIF, p-STAT3, MMP-2, MMP-9, VEGFA, and CyclinD1 were notably lower in the sh1-circSCAF8 group than in the control group (Fig. 9J, K and Supplementary Fig. 7A, B). Taken together, our results indicate that circSCAF8 has a significant role in the growth and metastasis of PCa cells in vivo, possibly via the LIF-STAT3 pathway.

## Discussion

Although circSCAF8 has been reported to be upregulated in PCa cells [7], the function of this circRNA in PCa and its underlying mechanisms have not been fully elucidated. In this study, we investigated the role and potential mechanisms of circSCAF8, which was significantly upregulated in PCa tissues and remarkably associated with the poor prognosis of PCa patients. Functionally, we found that the knockdown of circSCAF8 significantly suppressed cell proliferation, migration, and invasion abilities, as well as inhibited PCa oncogenesis and metastasis in vivo, while the overexpression of circSCAF8 had the opposite effects. Mechanistically, we showed that circSCAF8 can function as a sponge for both miR-140-3p and miR-335 to regulate LIF expression, which activated the LIF-STAT3 pathway, thereby leading to the growth and metastasis of PCa. Our study indicates that circSCAF8 may play a vital role in promoting PCa tumorigenesis and metastasis, and it could be used as a new diagnostic/prognostic marker and therapeutic target for PCa.

As a new member of the competing endogenous RNA (ceRNA) family, circRNAs have been reported to sponge miRNAs and interact with miRNAs to stabilize the miRNA-binding partner [7]. They mainly regulate the target gene that harbors the same miRNA-binding sites by competitively binding to miRNAs, thereby forming a complex post-transcriptional regulatory network [34]. For example, circTLK1 can bind to miR-136-5p to regulate CBX4 and VEGFA expression, thus promoting oncogenesis and the development of renal cell carcinoma [35]. CircPRKCI serves as a ceRNA to accelerate tumorigenesis of lung adenocarcinoma by binding to both miR-545 and miR-589, subsequently suppressing the downstream gene E2F7 [36]. Moreover, circMAT2B may function through absorbing miR-338-3p and upregulating its target gene PKM2 to promote hepatocellular carcinoma progression [37]. In the present study, we found that circSCAF8 was principally enriched in the cytoplasm of PCa cells. The bioinformatics analysis showed that circSCAF8 could sponge both miR-140-3p and miR-335. Subsequent RNA pull-down via biotin-coupled circSCAF8 probe and dual-luciferase reporter assays confirmed that circSCAF8 directly binds to both miR-140-3p and miR-335. Thus, our results suggest that circSCAF8 may act as an oncogene in PCa by sponging both miR-140-3p and miR-335.

Using the bioinformatics analysis, LIF was proposed as a target gene of both miR-140-3p and miR-335. Subsequently, we performed a dual-luciferase reporter assay to confirm the binding sites between these miRNAs and LIF. We found that upregulation of miR-140-3p or miR-335 was able to significantly suppress the expression level of LIF, while downregulation of miR-140-3p or miR-335 had the opposite effect. Other authors have reported that miR-140-3p could suppress tumorigenesis of squamous cell lung cancer by regulating the expression of BRD9 [38]. In addition, miR-335 could act as the bridge of lncRNA RP11-436H11.5 and BCL-W in regulating the proliferation and invasion of renal cell carcinoma [39]. Our in vivo metastasis models showed that knockdown of circSCAF8 significantly suppressed spontaneous lung metastases, while miR-140-3p inhibitors and miR-335 inhibitors partially reversed these effects.

LIF, a pleiotropic cytokine of the interleukin 6 families, participates in numerous vital biological processes, including cell differentiation, cancer metastasis, relapse, and drug resistance [40]. For example, in cholangiocarcinoma, LIF was found to regulate carcinogenesis and metastasis through the LIF/miR-181c pathway [41]. For triple-negative breast cancer, the enhanced level of LIF accelerates oncogenesis and progression by activating the LIF-STAT3 signaling pathway [42]. In the present study, LIF was found to be highly expressed in PCa tissues, and the abundance of LIF positively correlated with circSCAF8. In addition, we found that the downregulation of circSCAF8 decreased LIF expression at both mRNA and protein levels, while the upregulation of circSCAF8 increased LIF expression. However, the expression level of circSCAF8 in PCa cells was not affected by knockdown or overexpression of LIF. The phenotypic effects triggered by the silencing of circSCAF8 in PC3 cells were dampened by LIF overexpression. LIF inhibition could phenocopy the silencing of circSCAF8. Moreover, we confirmed that the upregulation of circSCAF8 led to the activation of the LIF-STAT3 signaling pathway, while the downregulation of circSCAF8 displayed opposite effects. Furthermore, miRNA (miR-140-3p and miR-335) mimics or inhibitors partially reversed these effects. Herein, our results verify the hypothesis that circSCAF8 could facilitate LIF expression and activate the LIF-STAT3 pathway by absorbing miR-140-3p and miR-335 to promote PCa progression.

Considering the clinical and translational significance of circSCAF8, both RT-PCR and FISH results showed that circSCAF8 is highly expressed in PCa tissues. Moreover, the high expression of circSCAF8 correlated with higher tumor stage, grade, and poor prognosis, thereby indicating that circSCAF8 may be a potential diagnostic/prognostic biomarker for PCa. Interestingly, we confirmed that the expression of circSCAF8 in urine extracellular vesicles was positively associated with that in PCa tissues. As a type of liquid biopsy assay, urine circSCAF8 measurements are repeatable, minimally invasive, and easily implemented during the course of treatment.

In summary, we confirmed that the novel circRNA, circSCAF8, has a profound role in tumorigenesis and metastasis of PCa. Mechanistically, circSCAF8 functions as a sponge for both miR-140-3p and miR-335 to regulate LIF expression and activate the LIF-STAT3 pathway. Our study suggests that circSCAF8 may become a potential biomarker and therapeutic target for PCa.

## Supplementary information

Supplemental figures and tables

Supplemental Material-uncropped original western blots

Reproducibility checklist

Certificate of English Language Editing

## Data Availability

All data generated or analyzed during this study are included in this published article and supplementary information files.

## References

[1] Sartor O, de Bono JS (2018). Metastatic prostate cancer. N Engl J Med.

[2] D’Amico AV, Chen MH, Roehl KA, Catalona WJ (2005). Identifying patients at risk for significant versus clinically insignificant postoperative prostate-specific antigen failure. J Clin Oncol.

[3] Freedland SJ, Humphreys EB, Mangold LA, Eisenberger M, Dorey FJ, Walsh PC (2005). Risk of prostate cancer-specific mortality following biochemical recurrence after radical prostatectomy. JAMA.

[4] Prensner JR, Zhao S, Erho N, Schipper M, Iyer MK, Dhanasekaran SM (2014). RNA biomarkers associated with metastatic progression in prostate cancer: a multi-institutional high-throughput analysis of SChLAP1. Lancet Oncol.

[5] Kreuz M, Otto DJ, Fuessel S, Blumert C, Bertram C, Bartsch S (2020). ProstaTrend-A multivariable prognostic RNA expression score for aggressive prostate cancer. Eur Urol.

[6] Vo JN, Cieslik M, Zhang Y, Shukla S, Xiao L, Zhang Y (2019). The landscape of circular RNA in cancer. Cell..

[7] Chen S, Huang V, Xu X, Livingstone J, Soares F, Jeon J (2019). Widespread and functional RNA circularization in localized prostate cancer. Cell..

[8] Prensner JR, Iyer MK, Balbin OA, Dhanasekaran SM, Cao Q, Brenner JC (2011). Transcriptome sequencing across a prostate cancer cohort identifies PCAT-1, an unannotated lincRNA implicated in disease progression. Nat Biotechnol.

[9] Du Z, Fei T, Verhaak RG, Su Z, Zhang Y, Brown M (2013). Integrative genomic analyses reveal clinically relevant long noncoding RNAs in human cancer. Nat Struct Mol Biol.

[10] He YD, Tao W, He T, Wang BY, Tang XM, Zhang LM (2021). A urine extracellular vesicle circRNA classifier for detection of high-grade prostate cancer in patients with prostate-specific antigen 2-10 ng/mL at initial biopsy. Mol Cancer.

[11] Liu Z, Zhou Y, Liang G, Ling Y, Tan W, Tan L (2019). Circular RNA hsa_circ_001783 regulates breast cancer progression via sponging miR-200c-3p. Cell Death Dis.

[12] Han D, Li J, Wang H, Su X, Hou J, Gu Y (2017). Circular RNA circMTO1 acts as the sponge of microRNA-9 to suppress hepatocellular carcinoma progression. Hepatology..

[13] Du WW, Yang W, Li X, Fang L, Wu N, Li F (2020). The circular RNA circSKA3 binds integrin β1 to induce invadopodium formation enhancing breast cancer invasion. Mol Ther.

[14] Xia X, Li X, Li F, Wu X, Zhang M, Zhou H (2019). A novel tumor suppressor protein encoded by circular AKT3 RNA inhibits glioblastoma tumorigenicity by competing with active phosphoinositide-dependent Kinase-1. Mol Cancer.

[15] Tan S, Kang Y, Li H, He HQ, Zheng L, Wu SQ (2021). circST6GALNAC6 suppresses bladder cancer metastasis by sponging miR-200a-3p to modulate the STMN1/EMT axis. Cell Death Dis.

[16] Han Z, Zhang Y, Sun Y, Chen J, Chang C, Wang X (2018). ERβ-mediated alteration of circATP2B1 and miR-204-3p signaling promotes invasion of clear cell renal cell carcinoma. Cancer Res.

[17] Gan X, Zhu H, Jiang X, Obiegbusi SC, Yong M, Long X (2020). CircMUC16 promotes autophagy of epithelial ovarian cancer via interaction with ATG13 and miR-199a. Mol Cancer.

[18] Xu H, Sun Y, You B, Huang CP, Ye D, Chang C (2020). Androgen receptor reverses the oncometabolite R-2-hydroxyglutarate-induced prostate cancer cell invasion via suppressing the circRNA-51217/miRNA-646/TGFβ1/p-Smad2/3 signaling. Cancer Lett.

[19] Yang Z, Qu CB, Zhang Y, Zhang WF, Wang DD, Gao CC (2019). Dysregulation of p53-RBM25-mediated circAMOTL1L biogenesis contributes to prostate cancer progression through the circAMOTL1L-miR-193a-5p-Pcdha pathway. Oncogene..

[20] Cao S, Ma T, Ungerleider N, Roberts C, Kobelski M, Jin L (2019). Circular RNAs add diversity to androgen receptor isoform repertoire in castration-resistant prostate cancer. Oncogene..

[21] Wang S, Chao F, Zhang C, Han D, Xu G, Chen G (2022). Circular RNA circPFKP promotes cell proliferation by activating IMPDH2 in prostate cancer. Cancer Lett.

[22] Yu YZ, Lv DJ, Wang C, Song XL, Xie T, Wang T (2022). Hsa_circ_0003258 promotes prostate cancer metastasis by complexing with IGF2BP3 and sponging miR-653-5p. Mol Cancer.

[23] Hansen EB, Fredsøe J, Okholm TLH, Ulhøi BP, Klingenberg S, Jensen JB (2022). The transcriptional landscape and biomarker potential of circular RNAs in prostate cancer. Genome Med.

[24] Ding L, Wang R, Shen D, Cheng S, Wang H, Lu Z (2021). Role of noncoding RNA in drug resistance of prostate cancer. Cell Death Dis.

[25] Shen Z, Zhou L, Zhang C, Xu J (2020). Reduction of circular RNA Foxo3 promotes prostate cancer progression and chemoresistance to docetaxel. Cancer Lett.

[26] Gao X, Pang J, Li LY, Liu WP, Di JM, Sun QP (2010). Expression profiling identifies new function of collapsin response mediator protein 4 as a metastasis-suppressor in prostate cancer. Oncogene..

[27] Piano MA, Brunello A, Cappellesso R, Del Bianco P, Mattiolo A, Fritegotto C (2020). Periostin and epithelial-mesenchymal transition score as novel prognostic markers for leiomyosarcoma, myxofibrosarcoma, and undifferentiated pleomorphic sarcoma. Clin Cancer Res.

[28] Sun QP, Li LY, Chen Z, Pang J, Yang WJ, Zhou XF (2010). Detection of TMPRSS2-ETS fusions by a multiprobe fluorescence in situ hybridization assay for the early diagnosis of prostate cancer: a pilot study. J Mol Diagn.

[29] Eisenhauer EA, Therasse P, Bogaerts J, Schwartz LH, Sargent D, Ford R (2009). New response evaluation criteria in solid tumours: revised RECIST guideline (version 1.1). Eur J Cancer.

[30] Chen LL (2016). The biogenesis and emerging roles of circular RNAs. Nat Rev Mol Cell Biol.

[31] Zheng Q, Bao C, Guo W, Li S, Chen J, Chen B (2016). Circular RNA profiling reveals an abundant circHIPK3 that regulates cell growth by sponging multiple miRNAs. Nat Commun.

[32] Yu CY, Li TC, Wu YY, Yeh CH, Chiang W, Chuang CY (2017). The circular RNA circBIRC6 participates in the molecular circuitry controlling human pluripotency. Nat Commun.

[33] Bartel DP (2009). MicroRNAs: target recognition and regulatory functions. Cell..

[34] Qu S, Liu Z, Yang X, Zhou J, Yu H, Zhang R (2018). The emerging functions and roles of circular RNAs in cancer. Cancer Lett.

[35] Li J, Huang C, Zou Y, Ye J, Yu J, Gui Y (2020). CircTLK1 promotes the proliferation and metastasis of renal cell carcinoma by sponging miR-136-5p. Mol Cancer.

[36] Qiu M, Xia W, Chen R, Wang S, Xu Y, Ma Z (2018). The circular RNA circPRKCI promotes tumor growth in lung adenocarcinoma. Cancer Res.

[37] Li Q, Pan X, Zhu D, Deng Z, Jiang R, Wang X (2019). Circular RNA MAT2B promotes glycolysis and malignancy of hepatocellular carcinoma through the miR-338-3p/PKM2 axis under hypoxic stress. Hepatology..

[38] Huang H, Wang Y, Li Q, Fei X, Ma H, Hu R (2019). miR-140-3p functions as a tumor suppressor in squamous cell lung cancer by regulating BRD9. Cancer Lett.

[39] Wang K, Jin W, Song Y, Fei X (2017). LncRNA RP11-436H11.5, functioning as a competitive endogenous RNA, upregulates BCL-W expression by sponging miR-335-5p and promotes proliferation and invasion in renal cell carcinoma. Mol Cancer.

[40] Jones SA, Jenkins BJ (2018). Recent insights into targeting the IL-6 cytokine family in inflammatory diseases and cancer. Nat Rev Immunol.

[41] Wang J, Xie C, Pan S, Liang Y, Han J, Lan Y (2016). N-myc downstream-regulated gene 2 inhibits human cholangiocarcinoma progression and is regulated by leukemia inhibitory factor/MicroRNA-181c negative feedback pathway. Hepatology..

[42] Zheng X, Huang M, Xing L, Yang R, Wang X, Jiang R (2020). The circRNA circSEPT9 mediated by E2F1 and EIF4A3 facilitates the carcinogenesis and development of triple-negative breast cancer. Mol Cancer.

