# Supplementary material for: CircSCAF8 promotes growth and metastasis of prostate cancer through the circSCAF8-miR-140-3p/miR-335-LIF pathway
Source: Cell Death Dis. 2022 Jun 2;13(6):517. doi: 10.1038/s41419-022-04913-7 (PMC9163066; doi:10.1038/s41419-022-04913-7)
Supplement: Supplementary file 1 — Supplemental figures and tables [file 41419_2022_4913_MOESM1_ESM.pdf]

CircSCAF8 promotes growth and metastasis of prostate cancer through the circSCAF8-miR-140-3p/miR-335-LIF pathway

Supplementary Figures and Tables

Figure S1

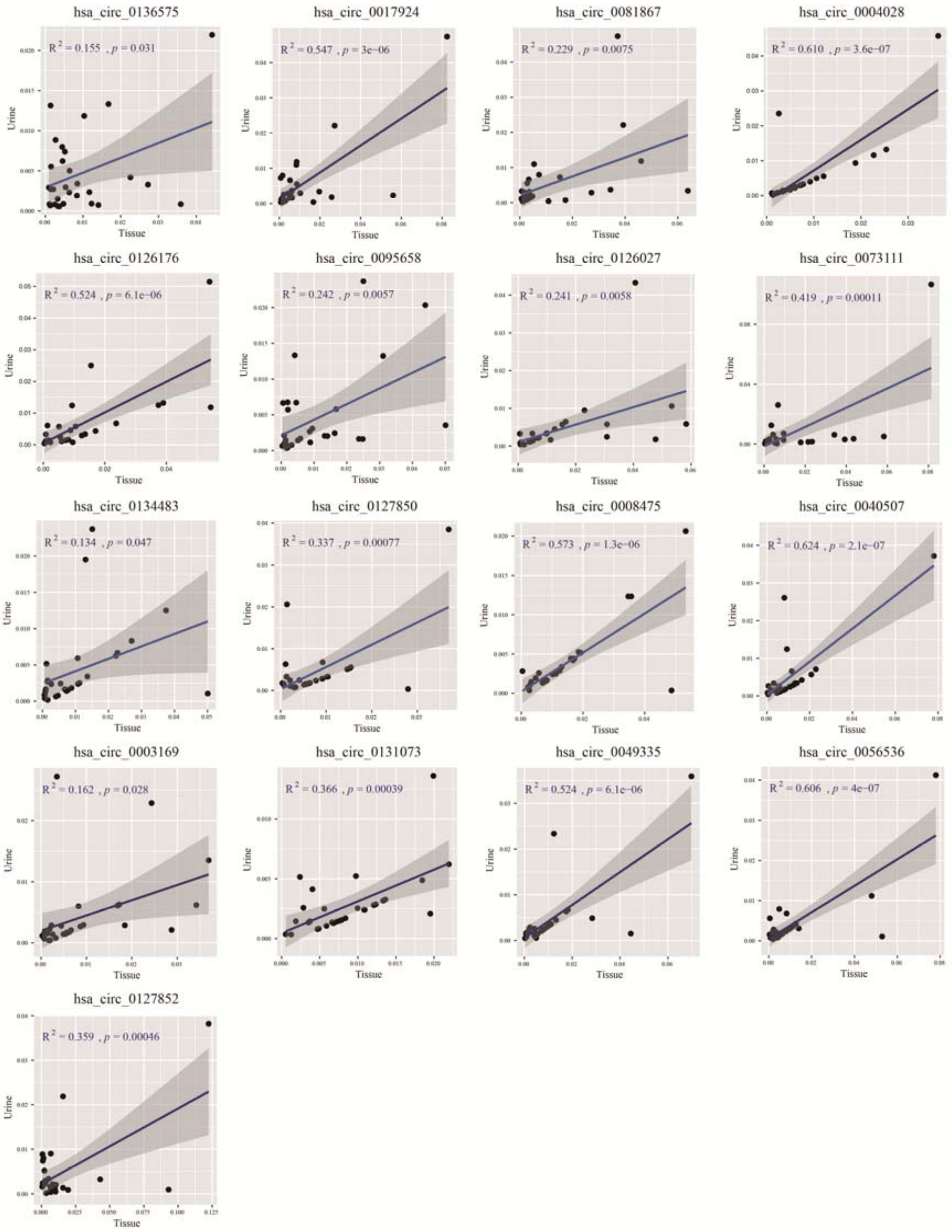

Figure S1. The Spearman correlation analysis of the expression levels of 17 circRNAs between the tumor tissue and matched urine extracellular vesicles in 30 PCa patients. The detail R^2 and P values were shown in the figure. Pearson's $\chi 2$ test was used to analyze statistical significance. PCa, prostate cancer; circRNAs, circular RNAs.

Figure S2A

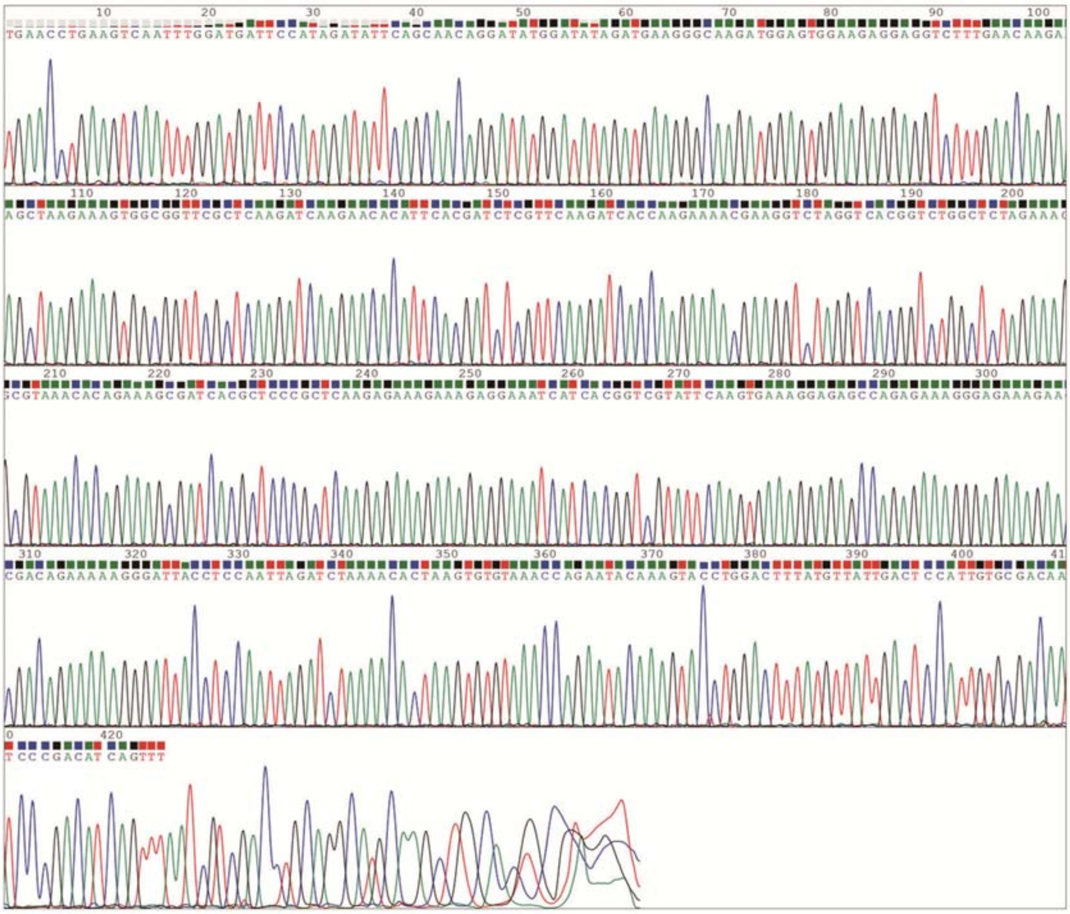

Figure S2B

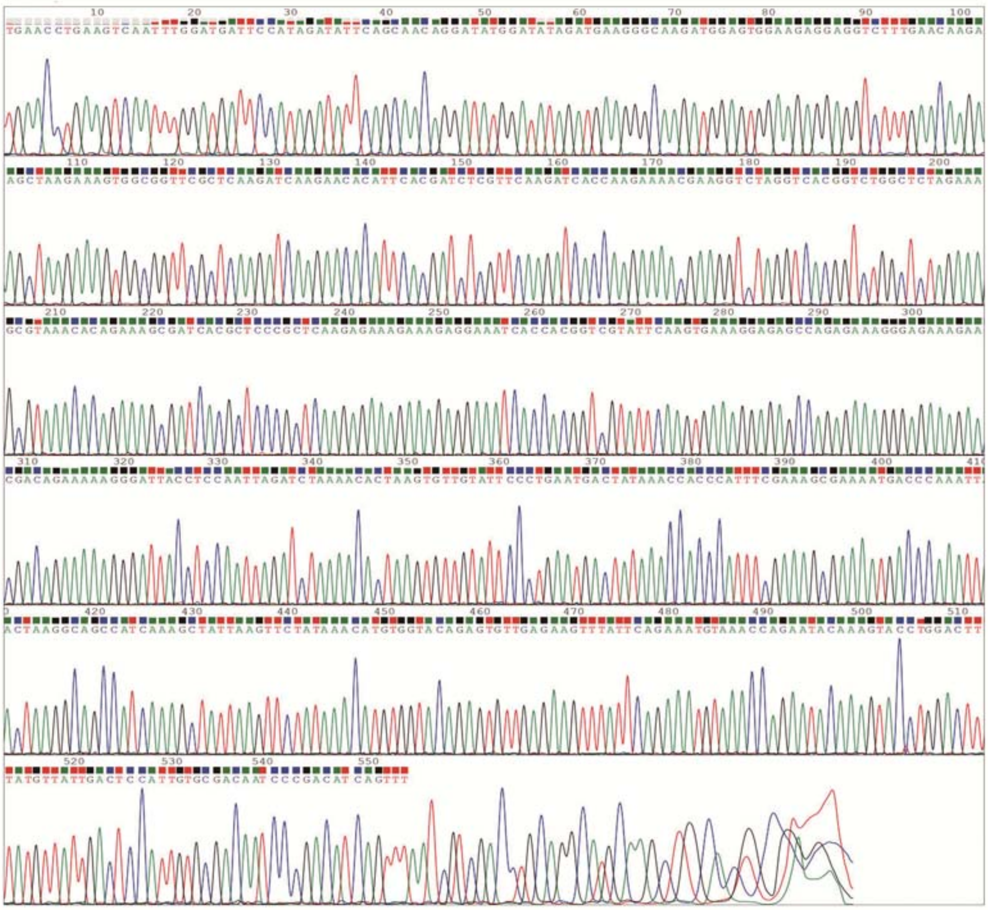

Figure S2C

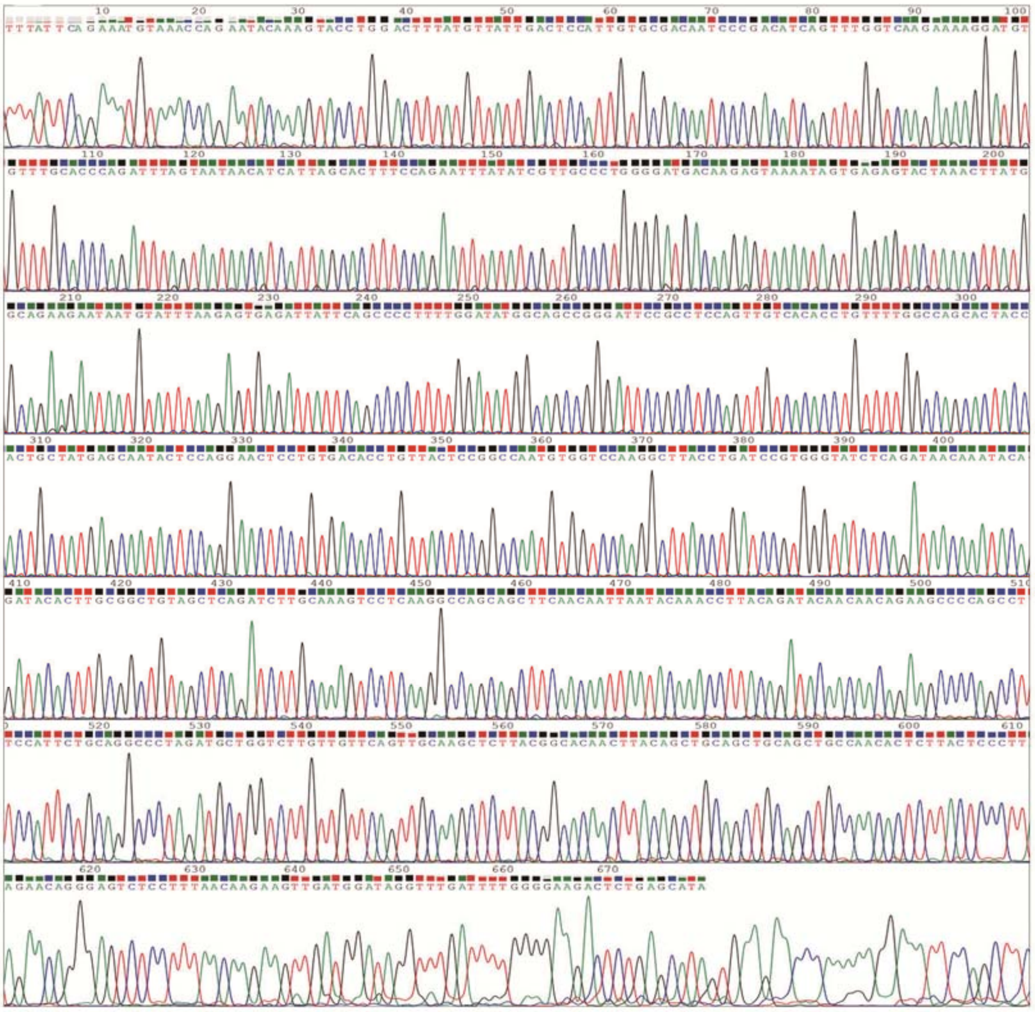

Figure S2D

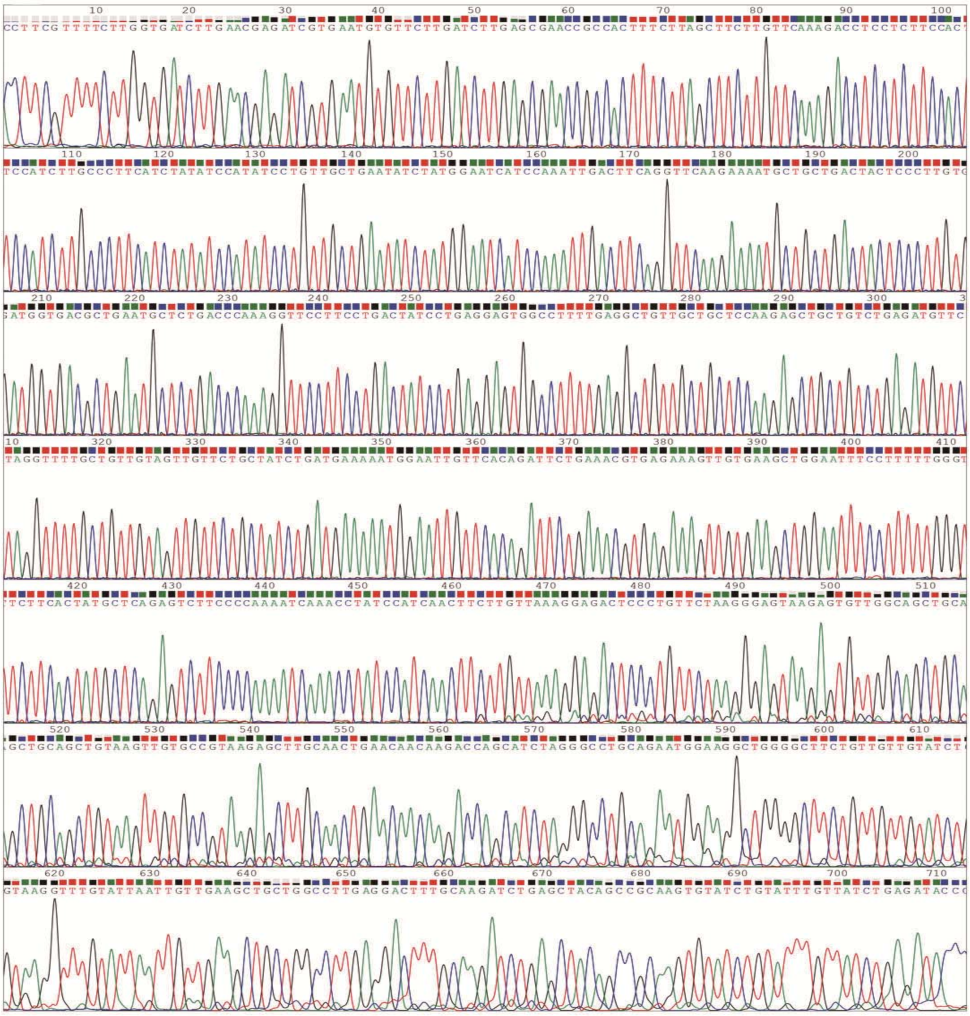

Figure S2 The rolling circle reverse transcription and Sanger sequencing were performed to detect the full length sequence of circSCAF8 in PC3 cells. The entire sequence of circSCAF8 was confirmed to be identical to that from circBase database. The results for rolling circle reverse transcription and Sanger sequencing from primer of RT-1038 F(**A**), RT-201 R(**B**), RT-64 F(**C**), RT-1269 R(**D**) were shown respectively.

Figure S3

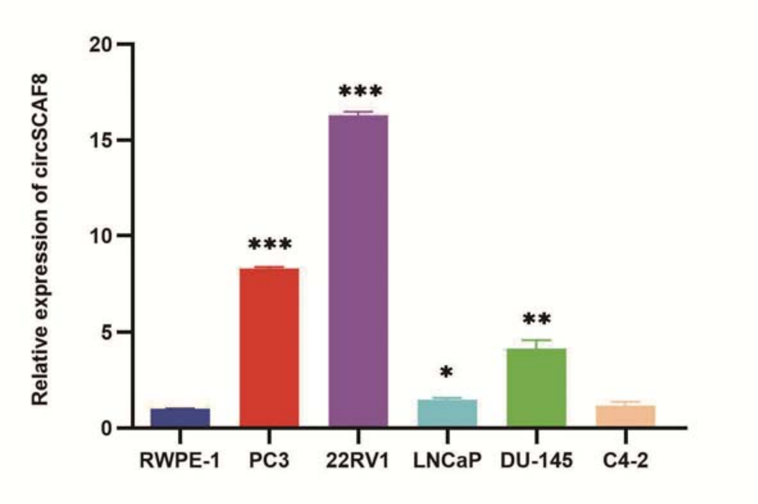

Figure S3 The results of qRT-PCR analysis of circSCAF8 expression in 5 human prostate carcinoma cell lines and one human prostate epithelial cell line, RWPE-1. All values were normalized to *GAPDH* and plotted relative to the expression of the RWPE-1 cell line. Error bars represent the standard deviation (SD) of three independent experiments. *, P<0.05; **, P<0.01; ***, P<0.001.

Figure S4

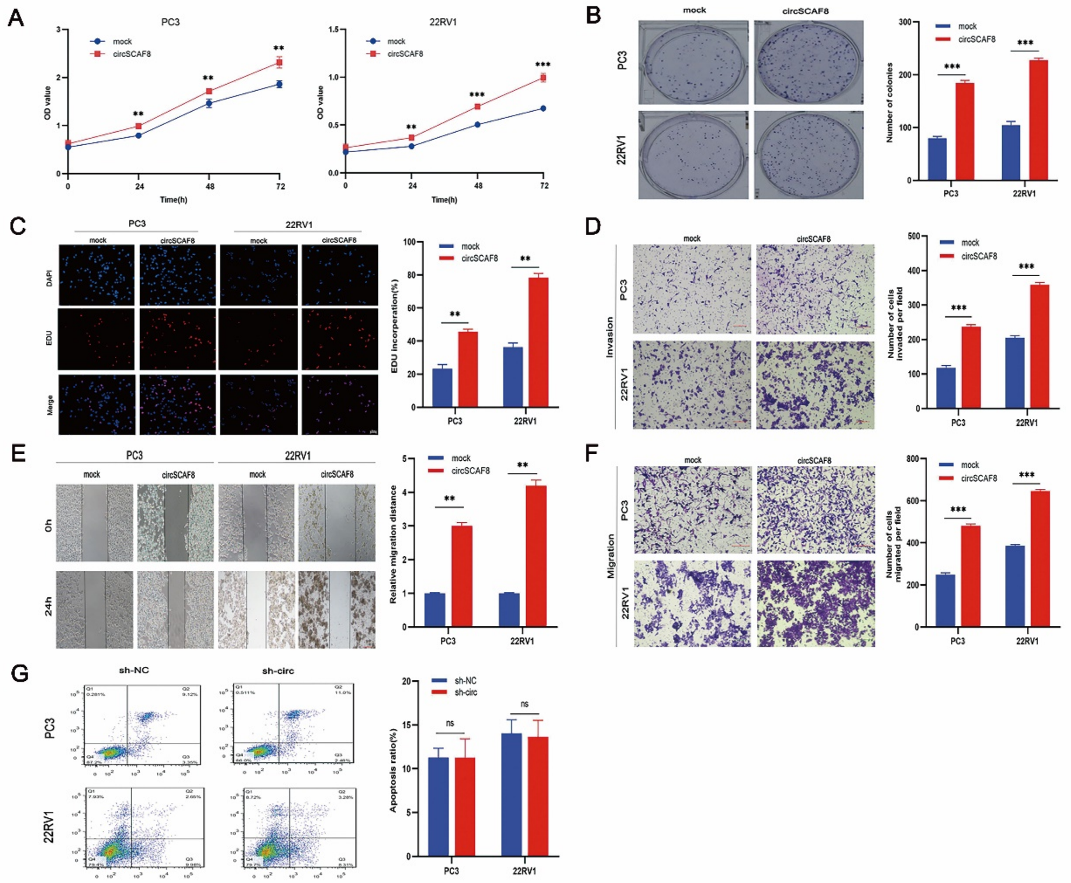

Figure S4 Upregulation of circSCAF8 accelerate the proliferation, invasion, and migration of PCa cells. **A-C** CCK8 (**A**), colony formation (**B**), and EDU (**C**) assays were implemented to test the proliferation ability of PC3 and 22RV1 cells, after upregulation of circSCAF8. **D** Transwell invasion assay was used to measure the invasion ability of PC3 and 22RV1 cells. **E** and **F** Wound healing and Transwell migration assays were carried out to assess the migration ability of PCa cells after upregulation of circSCAF8. **G** The apoptosis assay showed that there was no significant influence on PC3 and 22RV1 cells when downregulation of circSCAF8. Error bars represent the SD of three independent experiments. **, P<0.01; ***,P<0.001; ns, nonsignificant. PCa, prostate cancer; EDU, 5-ethynyl2'deoxyuridine.

Figure S5

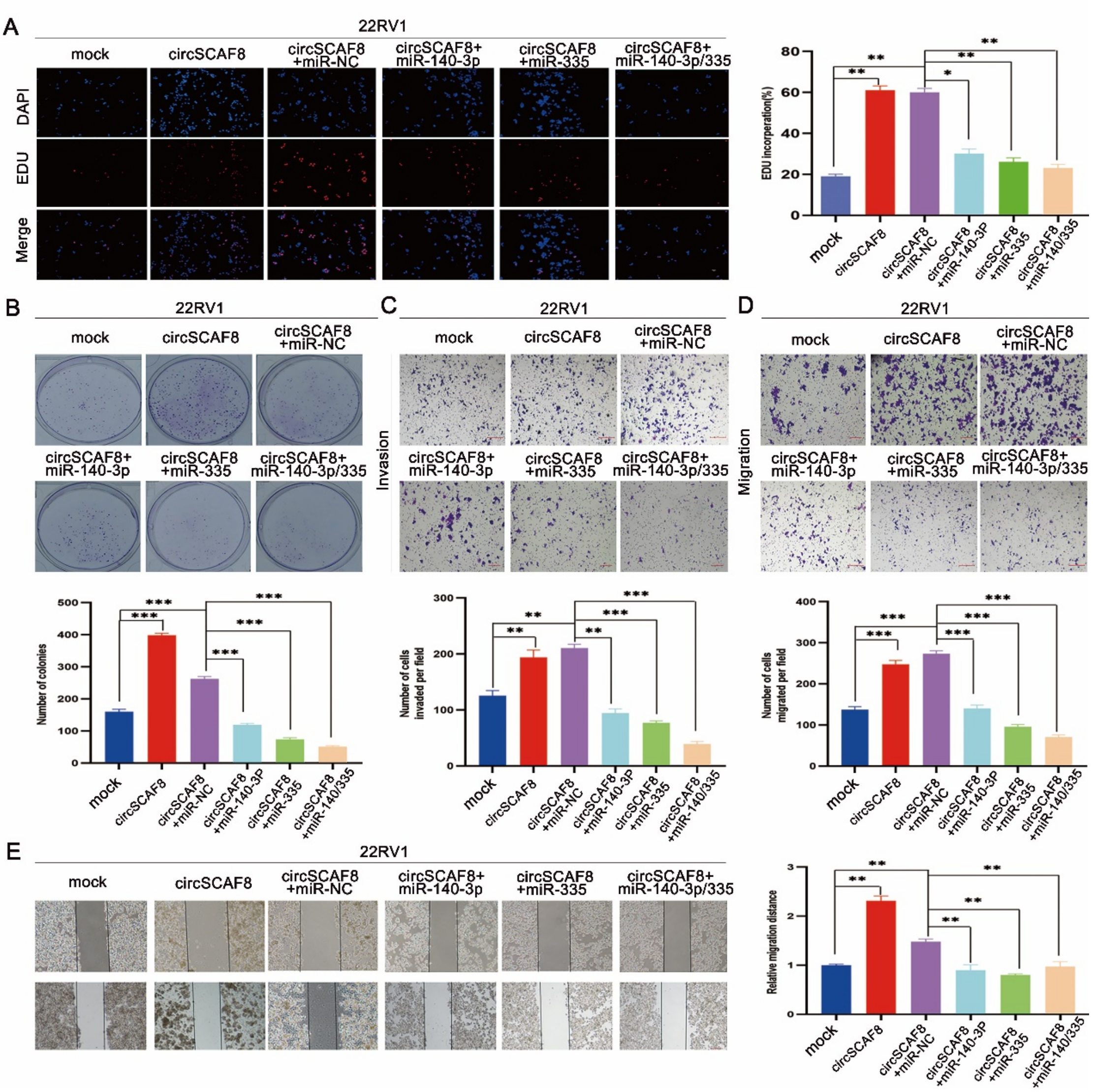

Figure S5 miR-140-3p mimics and miR-335 mimics could partially reverse the tumor-promoting effects caused by the upregulation of circSCAF8 in PCa cells. **A** and **B** The proliferation ability of 22RV1 cells with circSCAF8 upregulation treated with miR-140-3p mimics, miR-335mimics, and miR-140-3p/miR-335 mimics were measured by EDU (**A**) and colony formation assays (**B**). **C-E** Transwell invasion assay (**C**), transwell migration assay(**D**), and wound healing assay (**E**) were implemented to test the invasion and migration abilities of 22RV1 cells with circSCAF8 upregulation co-transfected with miR-140-3p mimics, miR-335 mimics, and miR-140-3p/miR-335 mimics. Error bars represent the SD of three independent experiments. *, $P< 0.05$; **, $P< 0.01$; ***, $P<0.001$. PCa, prostate cancer; EDU, 5-ethynyl2'deoxyuridine.

Figure S6

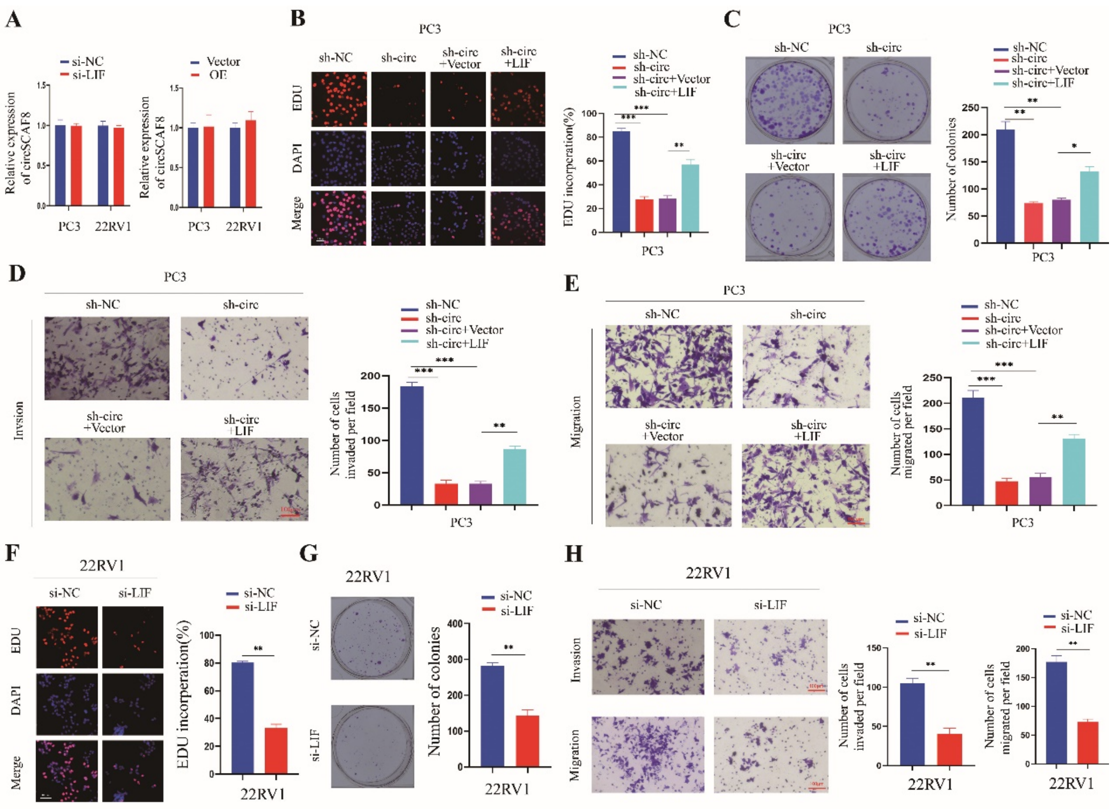

Figure S6 LIF could dampen the phenotypic effects triggered by the silencing of circSCAF8 in PCa cells. **A** The expression level of circSCAF8 after knockdown or overexpression of LIF in PC3 and 22RV1 cells. **B** and **C** The proliferation ability of PC3 cells with circSCAF8 knockdown co-transfected with LIF was measured by EDU (**B**) and colony formation assays (**C**). **D** and **E** Transwell invasion assay (**D**) and transwell migration assay (**E**) were implemented to test the invasion and migration abilities of PC3 cells with circSCAF8 downregulation co-transfected with LIF. **F** and **G** The proliferation ability of 22RV1 cells after knockdown of LIF was measured by EDU (**F**) and colony formation (**G**) assays. **H** The invasion and migration ability of 22RV1 cells after knockdown of LIF was determined by transwell assay. Error bars represent the SD of three independent experiments. *, P< 0.05; **,P< 0.01; ***, P<0.001. EDU, 5-ethynyl2'deoxyuridine.

Figure S7

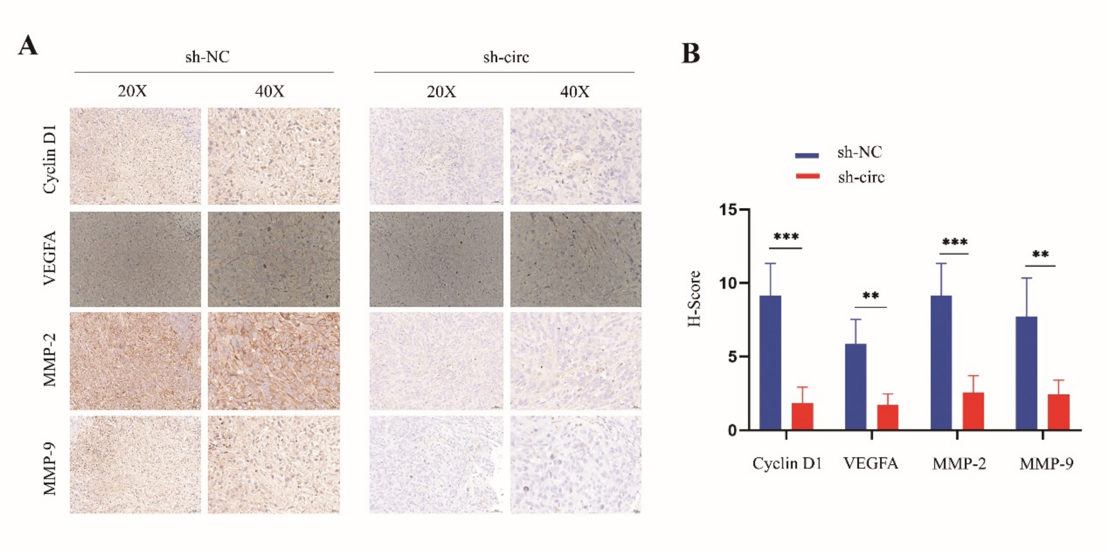

Figure S7 Representative images of IHC staining (**A**) and quantitative analyses **(B)** showing the intratumoural expression of Cyclin D1, VEGFA, MMP-2, and MMP-9 within orthotopic xenografts (n=7 for each group). **, P< 0.01; ***, P<0.001. IHC, immunohistochemistry.

Table S1 Sequences of shRNAs, si-RNA, probes, and primers used in this study

shRNAs, probes or primers	sequences
sh1-circSCAF8	5'- CTAAAACACTAAGTGTTGTAT-3'
sh2-circSCAF8	5'- CACTAAGTGTTGTATTCCCTG-3'
sh-NC	5'- TTCTCCGAACGTGTCACGT-3'
circSCAF8 probe (1)	5'-GGGAATACAACACTTAGTGT-3'
circSCAF8 probe (2)	5'-CAGGGAATACAACACTTAGT-3'
Oliogo probe	5'-AGTCGGAACTGAAATCATGT -3'
si-LIF	5'- CAACAACCUGGACAAGCUAUGUGGC-3'
GAPDH	F: 5'- CAGGAGGCATTGCTGATGAT-3'<br>R: 5'- GAAGGCTGGGGCTCATTT-3'
U6	RT:5'-GTCGTATCCAGTGCAGGGTCCGAGGTATTCGCACTGGATACGACAAAATA-3'<br>F: 5'- CTCGCTTCGGCAGCACA-3'<br>R: 5'- AACGCTTCACGAATTTGCGT-3'
SCAF8	F: 5'- GTCAGAGCATTCAGCGTCACC-3'<br>R: 5'- TCCACTCCATCTTGCCCTTC-3'
circSCAF8 (divergent)	F: 5'- TTGAAACTTTTGTCTTGGCTGG -3'<br>R: 5'- ATCTGCTGGCAATTCACTTTCT -3'
circSCAF8 (convergent)	F: 5'- CTGGCTCTAGAAAGCGTAAACACA-3'<br>R: 5'- TCCTTTCACTTGAATACGACCGT-3'
hsa-miR-140-3p	RT:5'-GTCGTATCCAGTGCGTGTCGTGGAGTCGGCAATTGCACTGGATACGACCCGTGGT -3'<br>F: 5'- CAGTGCTGTACCACAGGGTAGA -3'<br>R: 5'- TATCCTTGTTCACGACTCCTTCAC -3'
hsa-miR-335	RT:5'-GTCGTATCCAGTGCAGGGTCCGAGGTATTCGCACTGGATACGACACATTT-3'<br>F: 5'- CGCGTCAAGAGCAATAACGAA-3'<br>R: 5'- AGTGCAGGGTCCGAGGTATT-3'
hsa-miR-194-5p	RT:5'-GTCGTATCCAGTGCAGGGTCCGAGGTATTCGCACTGGATACGACTCCACA-3'<br>F: 5'- CGCGTGTAACAGCAACTCCA-3'<br>R: 5'- AGTGCAGGGTCCGAGGTATT-3'
hsa-miR-520f-3p	RT:5'-GTCGTATCCAGTGCAGGGTCCGAGGTATTCGCACTGGATACGACAACCCT-3'<br>F: 5'- CGCGAAGTGCTTCCTTTTAG-3'<br>R: 5'- AGTGCAGGGTCCGAGGTATT-3'
LIF	F: 5'- GGAACAGCAGAACTTAGGGTCA-3'<br>R: 5'- TCATGCCAGGTCAGACGCAC -3'
Rolling circle	RT-1038 F: 5'-TGAACCTGAAGTCAATTTGGATG -3'

amplification	RT-201 R: 5'- AAACTGATGTCGGGATTGTCG -3'
	RT-64 F: 5'- GCAGCCATCAAAGCTATTAAGTTC -3'
	RT-1269 R: 5'- GGAGCGTGATCGCTTTCTGT -3'

Table S2 Correlation between circSCAF8 expression and clinicopathologic characteristics of 85 patients with prostate cancer

Characteristics	circSCAF8 expression		
	low	high	P value
Age(y), No. (%)			0.920
<60	20 (51.3)	19 (48.7)	
≥60	22 (47.8)	24 (52.2)	
PSA (ng/mL), No. (%)			0.012
≤10	17 (73.9)	6 (26.1)	
>10	25 (40.3)	37 (59.7)	
Grade Group, No. (%)			0.038
≤GG2	28 (60.9)	18 (39.1)	
>GG2	14 (35.9)	25 (64.1)	
Pathologic stage, No. (%)			0.035
T1-2	30 (60.0)	20 (40.0)	
T3-4	12 (34.3)	23 (65.7)	
Lymph-node metastasis, No. (%)			0.444
Negative	34 (53.0)	31 (47.0)	
Positive	8 (40.0)	12 (60.0)	
