# Supplementary material for: CircSCAF8 promotes growth and metastasis of prostate cancer through the circSCAF8-miR-140-3p/miR-335-LIF pathway
Source: Cell Death Dis. 2022 Jun 2;13(6):517. doi: 10.1038/s41419-022-04913-7 (PMC9163066; doi:10.1038/s41419-022-04913-7)
Supplement: Supplementary file 2 — Supplemental Material-uncropped original western blots [file 41419_2022_4913_MOESM2_ESM.pdf]

CircSCAF8 promotes growth and metastasis of prostate cancer through the circSCAF8-miR-140-3p/miR-335-LIF pathway

Supplemental Material

Uncropped original western blots used in our manuscript

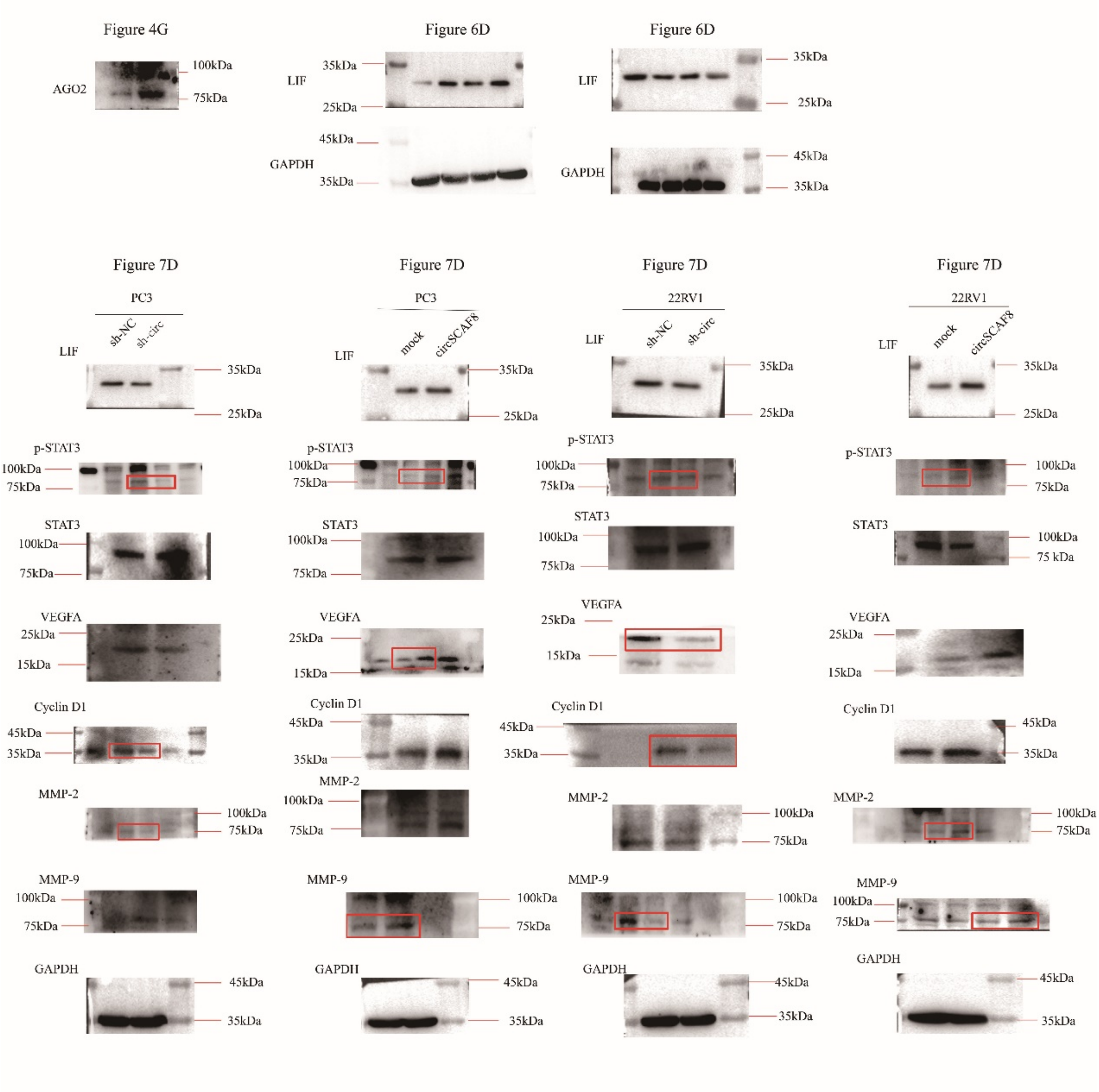

Figure S1 Uncropped western blots for Figure 4G, Figure 6D and Figure 7D

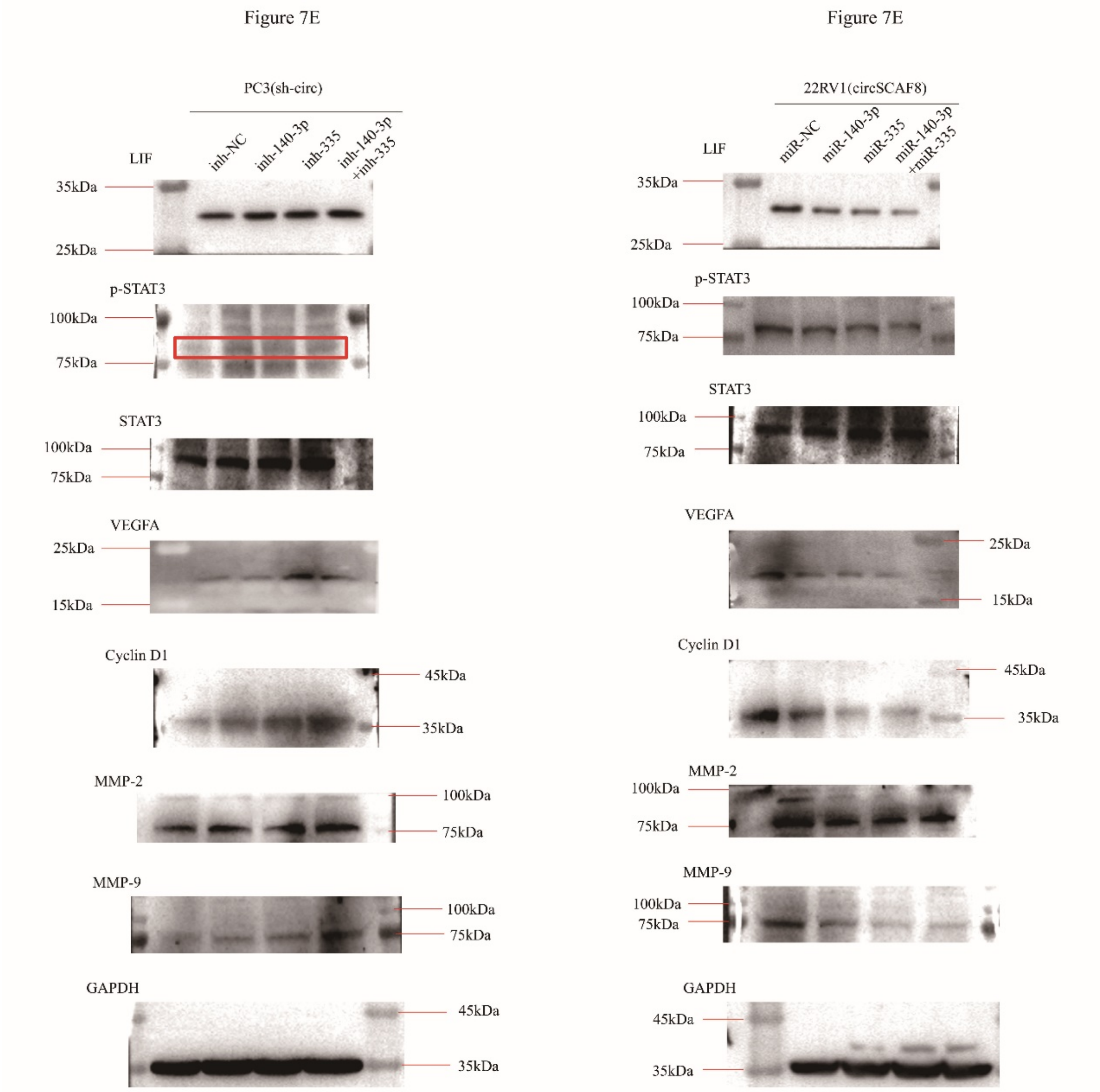

Figure S2 Uncropped western blots for Figure 7E
