# Supplementary material for: CircSCAF8 promotes growth and metastasis of prostate cancer through the circSCAF8-miR-140-3p/miR-335-LIF pathway
Source: Cell Death Dis. 2022 Jun 2;13(6):517. doi: 10.1038/s41419-022-04913-7 (PMC9163066; doi:10.1038/s41419-022-04913-7)
Supplement: Supplementary file 4 — Certificate of English Language Editing [file 41419_2022_4913_MOESM4_ESM.pdf]

Certificate of English Language Editing

Manuscript Title:

CircSCAF8 promotes growth and metastasis of prostate cancer through the circSCAF8-miR-140-3p/miR-335-LIF pathway

Date of Revision:

March 4, 2022

Abstract:
Circular RNAs (circRNAs) have been increasingly linked to cancer progression. However, the detailed biological functions of circRNAs in prostate cancer (PCa) remain unclear. Using high-throughput circRNA sequencing, we previously identified 18 urine extracellular vesicle circRNAs that were increased in patients with PCa compared with those with benign prostatic hyperplasia. Spearman correlation analysis of the expression levels of the 18 circRNAs between the tumor tissue and matched urine extracellular vesicles in 30 PCa patients showed that circSCAF8 had the highest R2 (R2 = 0.635, P < 0.001). The Cox proportional hazards regression model was used to estimate the effect of circSCAF8 on progression-free survival. The in vitro and in vivo functional experiments were implemented to investigate the effects of circSCAF8 on the phenotype of PCa. We found that the knockdown of circSCAF8 in PCa cells suppressed the proliferation, migration, and invasion ability, while overexpression of circSCAF8 had the opposite effects. Similar results were observed in vivo. In a cohort of 85 patients who had undergone radical...

This document certifies that the manuscript listed above was copy edited for English language by LetPub, with regard to grammar, punctuation, spelling, and clarity. All of our language editors are native English speakers with long-term experience in editing scientific and technical manuscripts. We are committed to leveling the playing field for researchers whose native language is not English.

- Documents receiving this certification should be regarded as having undergone professional editorial revision for English language before submission. However, the authors may accept or reject LetPub's suggestions and changes at their own discretion and LetPub does not have editorial control over the submitted documents.
- The language quality of the submitted document is the sole responsibility of the submitting authors subject to those authors' adherence to LetPub's revisions and instruction. LetPub's provision of service does not constitute a guarantee or endorsement of the authors' work herein.
- Neither the research content nor the authors' intended meaning were altered in any way during the editing process.
- If you have any questions or concerns about this edited document, please contact us at support@letpub.com

LetPub is an author service brand owned and operated by Accdon LLC. Headquartered in the Boston area, we are a full-spectrum author services company with a large team of US-based certified language and scientific editors, ISO 17001 accredited translators, and professional scientific illustrators and animators. We advocate ethical publication practices and are an official member of the Committee on Publication Ethics (COPE).

For more information about our company, services, and partnership programs, please visit www.letpub.com.

© 2022 Accdon, LLC. All Rights Reserved. Tel: 1-781-202-9968 Email: info@accdon.com Address: 400 Fifth Ave, Suite 530, Waltham, MA 02451, United States
